# Diagnostic and prognostic potential of the proteomic profiling of serum-derived extracellular vesicles in prostate cancer

**DOI:** 10.1038/s41419-021-03909-z

**Published:** 2021-06-21

**Authors:** Michele Signore, Romina Alfonsi, Giulia Federici, Simona Nanni, Antonio Addario, Lucia Bertuccini, Aurora Aiello, Anna Laura Di Pace, Isabella Sperduti, Giovanni Muto, Alessandro Giacobbe, Devis Collura, Lidia Brunetto, Giuseppe Simone, Manuela Costantini, Lucio Crinò, Stefania Rossi, Claudio Tabolacci, Marco Diociaiuti, Tania Merlino, Michele Gallucci, Steno Sentinelli, Rocco Papalia, Ruggero De Maria, Désirée Bonci

**Affiliations:** 1 RPPA Unit, Proteomics Area, Core Facilities, Istituto Superiore di Sanità, Rome, Italy; 2 Department of Oncology and Molecular Medicine, Istituto Superiore di Sanità, Rome, Italy; 3 IRCCS, Regina Elena National Cancer Institute, Rome, Italy; 4 Dipartimento di Medicina e Chirurgia Traslazionale, Università Cattolica del Sacro Cuore Largo F. Vito 1, 00168 Rome, Italy; 5 Fondazione Policlinico Universitario A. Gemelli IRCCS, Roma, Italy; 6 Department of Urology, Humanitas University, Turin, Italy; 7 Department of Urology, S. Giovanni Bosco Hospital, Turin, Italy; 8 Department of Urology–IRCCS Regina Elena National Cancer Institute of Rome, Rome, Italy; 9 Department of Oncology, IRST-Meldola, Meldola, Italy; 10 Department of Rare Diseases, Istituto Superiore di Sanità, Rome, Italy; 11 Department of Urology, Sapienza University of Rome, Rome, Italy; 12 Department of Urology Campus Biomedico, Rome, Italy

**Keywords:** Tumour biomarkers, Protein-protein interaction networks

## Abstract

Extracellular vesicles (EVs) and their cargo represent an intriguing source of cancer biomarkers for developing robust and sensitive molecular tests by liquid biopsy. Prostate cancer (PCa) is still one of the most frequent and deadly tumor in men and analysis of EVs from biological fluids of PCa patients has proven the feasibility and the unprecedented potential of such an approach. Here, we exploited an antibody-based proteomic technology, i.e. the Reverse-Phase Protein microArrays (RPPA), to measure key antigens and activated signaling in EVs isolated from sera of PCa patients. Notably, we found tumor-specific protein profiles associated with clinical settings as well as candidate markers for EV-based tumor diagnosis. Among others, PD-L1, ERG, Integrin-β5, Survivin, TGF-β, phosphorylated-TSC2 as well as partners of the MAP-kinase and mTOR pathways emerged as differentially expressed endpoints in tumor-derived EVs. In addition, the retrospective analysis of EVs from a 15-year follow-up cohort generated a protein signature with prognostic significance. Our results confirm that serum-derived EV cargo may be exploited to improve the current diagnostic procedures while providing potential prognostic and predictive information. The approach proposed here has been already applied to tumor entities other than PCa, thus proving its value in translational medicine and paving the way to innovative, clinically meaningful tools.

## Introduction

Prostate cancer (PCa) is still the second cause of cancer-related male deaths in highly developed countries [1]. A significant fraction of PCa patients arrives at diagnosis with advanced forms, while others retain indolent tumors which will never progress into aggressive stages [2, 3]. Therefore, an accurate, early diagnosis is likely to improve the outcome and the quality of life of PCa patients while reducing the over-treatment [4].

Extracellular vesicles (EVs) are membrane-enclosed bodies in the nano- to micro-meter scale that are secreted by nearly all cells and shuttle their biological content as a means of cell-to-cell communication [5, 6]. Tumor cells are now recognized to release more EVs than their normal counterpart and tumor-derived EVs can be easily isolated from bodily fluids [7–10], thus offering an exquisite source in terms of biomarkers and, mechanistically, of cancer treatment strategies [11–13]. The EV sub-population in the range of 30–150 nm in diameter is referred to as exosomes and has been shown to actively transport DNA, proteins, long and small RNAs [11, 14] as well as small peptides, such as prions [15]. Different from other vesicles, which are generated by random shedding mechanisms or from dying cells by discharge, exosomes drive intra- and inter-tissue cross-talk [16–18], are involved in physiological tissue homeostasis and immune system regulation [11] and in processes [12, 19, 20] that are often aberrant in tumors [7]. In this regard, PCa is characterized by multiple genomic lesions [21] and several variants seem to be associated with tumor development [22, 23]. Therefore, the EV cargo in PCa patients could be a promising source of new biomarkers [24] that deserve intense investigation.

The importance of better classifying patients into risk-progression categories has given impetus to the identification of prognostic biomarkers [21]. However, the available diagnostic tools for PCa therapy remain far from satisfying. The application of mass spectrometry-based proteomics has provided insights into the clinical management of PCa [25]. However, the limitations, in terms of sensitivity, throughput and clinical applicability of mass spectrometry, leave margins for other methodologies [25–27].

Here, by using an antibody-based technique, i.e. the Reverse-Phase Protein microArrays (RPPA), we evaluated the proteomic content of EVs isolated from blood sera of PCa tumors, healthy donors, patients with hypertrophic disease, and post-prostatectomy disease-free cases. A retrospective cohort with 15-year follow-up was further included for defining the prognostic value of biomarkers. Our results confirm and expand upon the concept that EVs shuttle cancer-related biomarkers and provide a useful platform for diagnostic as well as prognostic purposes. The combined proteomic and EV-based liquid-biopsy approaches used herein advocate for the use of new, non-invasive cancer monitoring tools in the personalized treatment of PCa.

## Results

### Use of RPPA technology as a proteomic approach to analysis of EV cargo

**Fig. 1:**
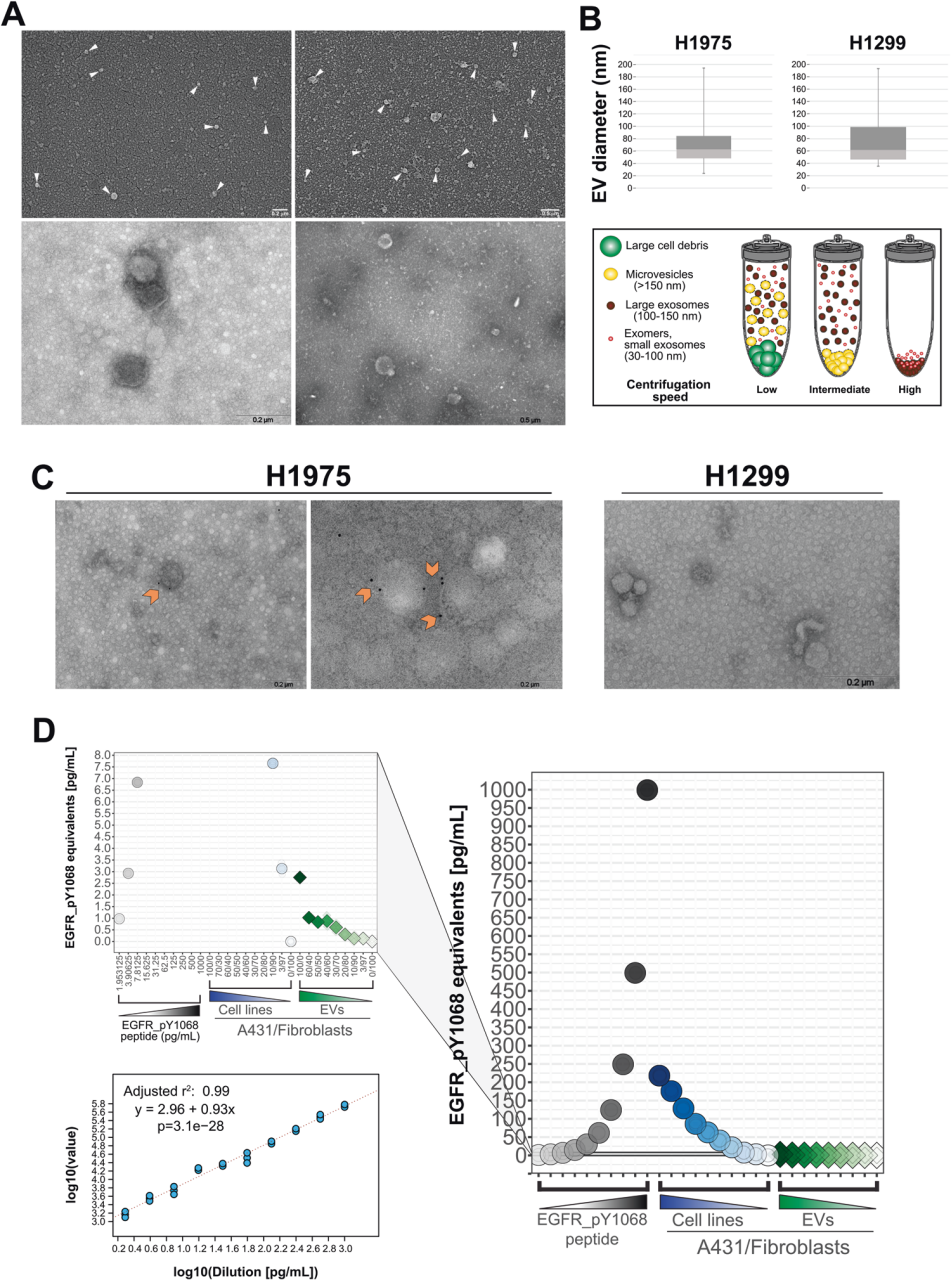
Isolation and RPPA testing of cell line-derived EVs. **A** Representative images of EVs purified from conditioned medium of H1975 and H1299 cells by Scanning and Transmission Electron Microscopy (SEM and TEM) analyses, upper and lower panels, respectively. The images shown are representative of all cell lines used in the article. **B** Boxplots representative of H1975 and H1299 EVs size distribution evaluated by SEM analysis. The lower schematic representation exemplifies the size distribution of EVs. **C** EpCAM antigen evaluated in EVs purified from cultures of H1975 and H1299 by Immuno-Electron Microscopy (IEM). **D** RPPA measurement of absolute amounts of EGFR_pY1068 in A431 and fibroblasts. Whole cell and EV lysates were first diluted to a total protein concentration of 0.5 mg/ml and then printed as mixtures with the indicated decreasing and increasing fractions of A431 and fibroblasts, respectively. A dilution curve of EGFR_pY1068 synthetic peptide in the plotted range, was printed along with cell line and EV samples and provided a reference curve (lower-left panel) to predict absolute concentrations of EGFR_pY1068 in A431 and fibroblast mixtures (upper panel). Data in the left side of the upper panel represent a magnification of the low picogram range from the adjacent (right) scatterplot. Main points represent the mean of technical replicates (*n* = 3, empty symbols behind each main-colored point). The reference curve in the lower panel has been used to predict EGFR_pY1068 absolute levels in samples via simple linear regression (red line) of log10-converted triplicates of normalized RPPA levels from the EGFR_pY1068 synthetic peptide dilution curve. Adjusted r squared, regression formula and p-value are shown inside the reference curve plot.

We and others have already shown that the RPPA technology is a powerful biomarker discovery platform for the analysis of EV protein cargo [28, 29]. Therefore, we decided to leverage on our previous findings to further investigate the analytical performance of a combined RPPA-EV approach. To this end, we first selected suitable cell line models and isolated EVs by differential ultracentrifugation of conditioned culture medium (Fig. S1A). Analysis by Scanning and Transmission Electron Microscope (SEM and TEM, respectively) showed that the majority (90%) of enriched EVs ranged from 34 to 100 nm (Fig. 1A, B). RPPA and Western Blotting analyses showed that such isolated EVs expressed transmembrane and cytosolic proteins that are typically enriched in exosomes [30] (Fig. S1B, C). As a specificity test we selected the epithelial cell adhesion molecule (EpCAM) [31, 32] since it is a reference marker in circulating cancer cell capture approaches and is used to search for disseminated tumor cells in bone marrow biopsies [33]. In this regard, we used two lung cancer cell lines, i.e. H1975 and H1299, that are positive and negative for EpCAM, respectively [34, 35] (Fig. S1D). Western blotting and TEM Immunogold labeling confirmed that EVs isolated from H1975 and H1299 paralleled the expression of EpCAM in their parent cells (Fig. S1E and Fig.1C). In order to test the limit of detection of the RPPA platform, we measured EpCAM in scaling mixtures of H1299- and H1975-derived EVs. EpCAM RPPA levels positively correlated with the percentage of H1975-derived EVs and were still detectable over the background down to as low as 3% of H1975 EVs (Fig. S1F). Furthermore, we selected HT29, an EpCAM^+^ colon cancer line [36] (Fig. S1G) and the metastatic cell lines LNCaP and PC3, positive and negative for EpCAM, respectively (Fig. S2A). Similar to what we found in lung cancer cell lines, we successfully measured EpCAM by RPPA in scaling mixtures of EVs isolated from either HT29 and H1299 (Fig. S1H) or LNCaP and PC3 (Fig. S2B), both showing detectable expression over the background down to 3% of positive EVs. To expand upon the concept of using RPPA as a sensitive biomarker discovery platform for low-abundance antigens or proteins expressed by low-frequency cancer sub-clones [37], we studied the expression of two markers, IL-6 and TGF-β, reportedly shuttled in EVs and over-expressed in advanced cancers [6, 24]. TGF-β and IL-6 were differentially expressed in PC3 and LNCaP cells (Fig. S2A), and resulted undetectable in EVs by Western blotting (data not shown). Conversely, RPPA analysis of LNCaP- and PC-derived EVs, mixed in scaling percentages, showed that TGF-β and IL-6 expression in EVs mirror the pattern found in parent cells (Fig. S2C, D). Notably, when measuring TGF-β by RPPA in EVs isolated from PC3 cells along with a cytokine reference standard, we were able to detect down to as low as 7 pg/mL of TGF-β in PC3-derived EVs (Fig. S2D).

**Fig. 2:**
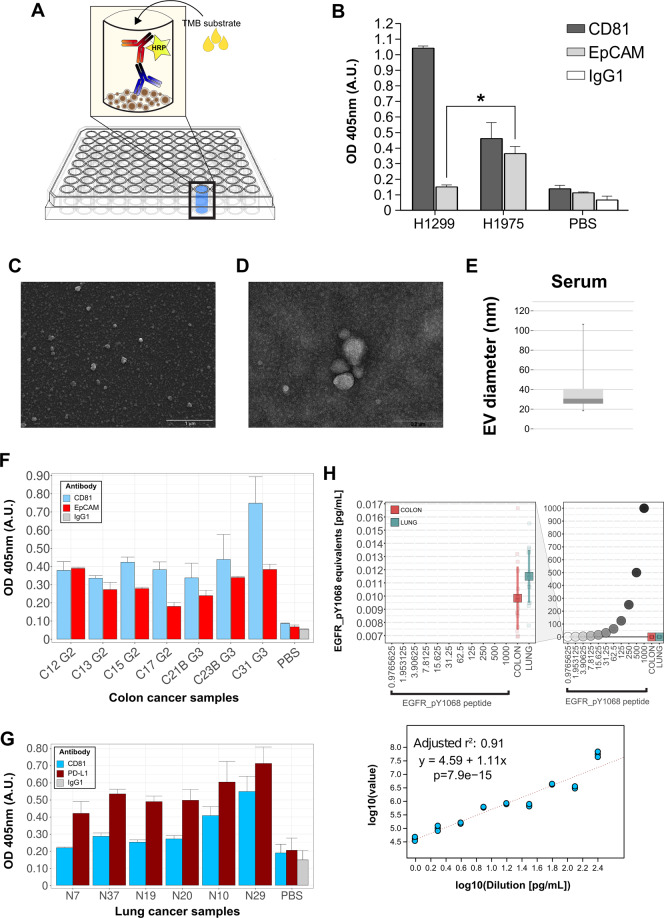
Orthogonal validation of RPPA results on serum-derived EVs. **A** Representative scheme of our homemade ELISA test, i.e. ELEXO, including EV-coated microtiter plate, primary antibody, HRP-conjugated secondary antibody and colorimetric reaction with TMB substrate. **B** H1975 and H1299 EVs analyzed by ELEXO assay. IgG isotype and Phosphate-Buffered Saline (PBS) were used as internal controls. Data are reported as mean and SD (*n* = 3) of arbitrary units of O.D. (Optical Density) at the specified wavelength (nm) (*p = 0.02). **C**, **D** Representative images of SEM (**C**) and TEM (**D**) analysis of EVs isolated from the sera of prostate cancer patients. Images are representative of pivotal and training cohort PCas. **E** Box plot representative of serum EV size distribution evaluated by SEM analysis. **F**, **G** ELEXO measurement of EpCAM (**F**) and PD-L1 (**G**) antigens in EVs isolated from colon and lung cancer patients, respectively. CD81 has been used as endogenous EV control. Data are reported as mean and SD (*n* = 3) of arbitrary units of O.D. (Optical Density) at the specified wavelength (nm). Internal reference controls were reported as IgG, CD-81, EpCAM, or PD-L1 antibody in PBS condition. **H** Absolute RPPA quantification of EGFR_pY1068 synthetic peptide in dilution curve along with isolated EVs from sera of colon and lung cancer patients. The expression levels in EVs fall below the lowest EGFR_pY1068 peptide dilution point and their scale is magnified in the left panel of the plot. The reference curve in the lower panel was obtained as in Fig. 1D to predict absolute EGFR_pY1068 RPPA levels in EV samples.

Besides merely testing the analytical performance of RPPA, we were particularly interested in measuring post-translational modifications, e.g. phosphorylation, of proteins shuttled by EVs. To this end, we evaluated the expression of the epithelial-growth factor receptor (EGFR) and one of its phosphorylated forms (EGFR_pY1068) in A431, an epithelial squamous carcinoma cell line, and in human PCa-derived activated fibroblasts (CAFs). Indeed, EGFR is expressed at high levels by A431 (1–2 ×10^6^ receptors per cell [38, 39]) while being low to undetectable in fibroblasts [40]. EVs were isolated from culture media and mixed in scaling percentages for RPPA analysis. As expected, the levels of EGFR were proportional to the percentage of A431 in each mixture point (Fig. S2E) while those of PD-L1, reportedly expressed by both A431 and CAFs [41, 42], did not follow a dilution curve (Fig. 2E). In line with the expression of its total content counterpart, RPPA levels of phosphorylated EGFR (EGFR_pY1068) positively correlated with the fraction of A431 in mixed A431/CAFs EVs (Fig. S2F). Driven by these results, we sought to quantify the levels of EGFR_pY1068 in scaling mixtures of A431 and CAFs as well as of their isolated EVs using a reference curve of a synthetic EGFR_pY1068 peptide, ranging from the nanogram down to the picogram scale. Interestingly, although cell-derived extracts displayed significantly higher amounts, RPPA expression of EGFR_pY1068 was still detectable below the picogram range in sample mixtures with increasing fractions of A431-derived EVs (Fig. 1D). Even though the sensitivity and specificity may depend on both the individual antibody performance and antigen relative expression, our data soundly suggest that the proposed approach is feasible and robust.

### Validation of RPPA results by an ELISA-based assay

In order to validate and integrate RPPA with orthogonal approaches, we set up a homemade ELISA (enzyme-linked immunosorbent assay), hereafter referred to as ELEXO. In such an assay, EVs are directly coated to the plastic plate and are subsequently probed for the expression of different surface markers by using primary and HRP-conjugated secondary antibodies followed by colorimetric detection (Fig. 2A). Thus, we took advantage of ELEXO to measure EpCAM and the exosomal marker CD81 on EVs isolated from H1975 and H1299. Similar to our previous RPPA and Western blot analyses, EpCAM levels were significantly detectable over the background only in H1975-derived EVs (Fig. 2B). EVs isolated from HT29 and SW480 colorectal cancer cell lines express CD81 and display differential levels of EpCAM by Western Blotting (Fig. S3A) as well as by ELEXO (Fig. S3B). Subsequently, we focused on the clinically relevant antigen PD-L1 [43] and, in order to test the antibody specificity, we transduced 293T cells with either empty (TWEEN) or PD-L1 gene (PD-L1) lentiviral vectors, respectively. We then isolated EVs from relative culture media and successfully measured PD-L1 by ELEXO only on those deriving from PDL-1-transduced cells (Fig. S3C). In addition, since both H1975 and H1299 cells express PD-L1 (Fig. S3D) we used ELEXO to confirm the presence of PD-L1 on their secreted EVs (Fig. S3E). Similar results were obtained by RPPA analysis of PD-L1 levels on a series of matched cells and cell-derived EVs including H1975 and H1299 samples (Fig. S3F).

Encouraged by these results, we envisioned an EV-based liquid-biopsy approach [44] built on RPPA analysis of EVs isolated from cancer patients’ serum samples. The majority of isolated vesicles (85%) showed sizes ranging between 34 and 100 nm as by SEM and TEM analyses (Fig. 2C–E and Fig. S4A). Analysis of typical exosome markers [45–47] confirmed their expression in EVs derived from sera of a small set of healthy donors, hypertrophic disease and PCa samples by Western blotting (Fig. S4B). Similarly, we measured exosomal proteins by RPPA in EVs isolated from a set of 12 PCa samples (Fig. S4C). Then we used ELEXO to evaluate EpCAM levels in EVs isolated from the serum of 7 colon adenocarcinoma (CRC) samples (Table S1). Our results showed that EpCAM is not only expressed by colon tumors [31, 32], but also a potential biomarker for CRC-derived EVs (Fig. 2F). Since PD-L1 is a predictor biomarker of immunotherapy response in advanced Non-Small-Cell lung cancer (NSCLC) [48], we sought to apply ELEXO to detect PD-L1 in serum EVs isolated from 6 NSCLC samples (Table S1, Fig. 2G), thus confirming the clinical applicability of ELEXO. Parallel to the above experimental design on cell line models, we tested the possibility to use RPPA to measure, via a reference dilution curve, the levels of EGFR_pY1068 in EVs isolated from the serum of primary NSCLC and CRC samples (*n* = 35) (Table S1). Our results show that, although not quantifiable, EGFR_pY1068 is still detectable above the background even below the picogram level (Fig. 2H) suggesting the opportunity for accurate measurement of phosphorylated and non-phosphorylated analytes in serum-derived EVs.

### Pivotal analysis on a cohort of PCa patients

**Fig. 3:**
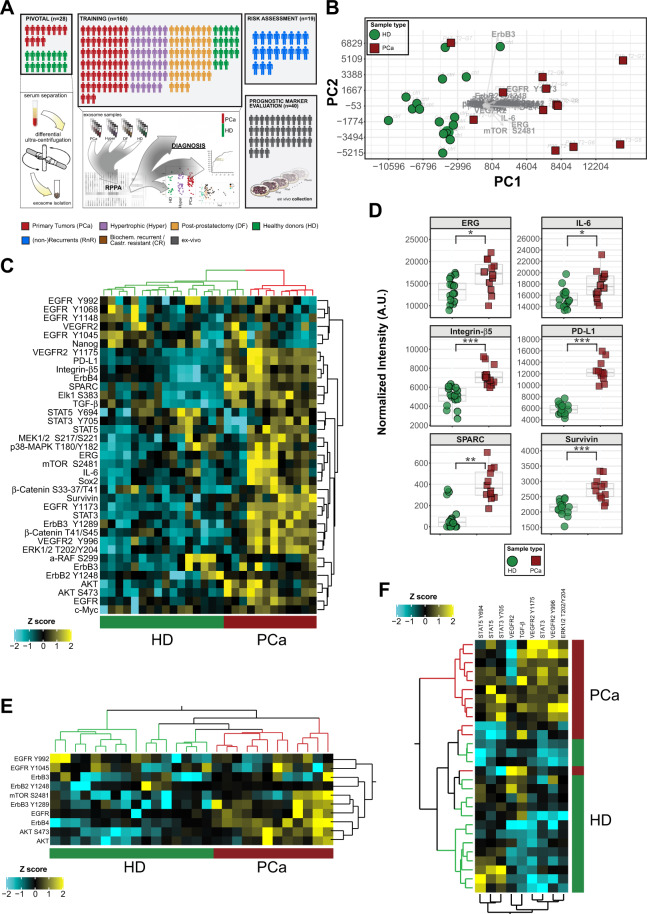
RPPA study sets and results on the pivotal cohort. **A** Schematic representation of the experimental study design. The cohorts assayed comprise a (i) pivotal group of primary prostate cancer (PCa) and healthy donor (HD) samples that have been utilized for experimental setup, i.e. EV isolation and RPPA sensitivity tests, (ii) training cohort including PCa, hypertrophic (Hyper), post-prostatectomy-disease-free (DF) cases as well as healthy donor (HD) EV samples and used to confirm and expand upon the RPPA analysis of the pivotal cohort in search of diagnostic markers, iii) a set of two independent cohorts of PCa samples used for risk assessment and prognostic marker evaluation, respectively. The risk assessment cohort comprises samples with 15-year documented follow-up (recurrent and non-recurrent). The prognostic marker evaluation cohort is composed of forty primary cell lines established from patients with bad and good documented prognosis [66]. **B** Principal component analysis (PCA) of 37 RPPA endpoints measured in EV samples from the pivotal cohort (16 healthy donor, HD, and 12 tumors, PCa). PCA algorithm employed the covariance matrix obtained from normalized RPPA intensity values. Scores (i.e. samples) are represented as dots (HD, green) and squares (PCa, red) while loadings (i.e. RPPA antibodies) are overlaid and pointed by gray arrows. **C** Two-way unsupervised hierarchical clustering of the same dataset as in (**B**). Normalized RPPA intensity values were pre-standardized (Z score) and the color intensity scale indicates high (yellow), average (black) and low (cyan) relative expression. **D** Scatterplots of selected statistically significant RPPA endpoints, resulting from comparison of HD- and PCa-derived EVs. Statistical significance by Student’s *t* test is reported on each plot and coded with asterisk(s) based on the level of significance (*p < = 0.05, **p < = 0.01, ***p < = 0.001). **E**, **F** Two-way unsupervised hierarchical clustering on selected panels of significant RPPA endpoints (*p* < = 0.05) associated with growth factor receptor signaling and angiogenesis (respectively **E**, **F**). Statistical significance was calculated by comparing normalized RPPA intensity values of HD and PCa, as in (**D**). Hierarchical clustering was performed on standardized RPPA data as in (**C**) and Z scores are color-coded as high (yellow), average (black) and low (cyan).

Inspired by the results obtained by RPPA and ELEXO on EVs derived from cell lines and cancer patients’ sera, we designed a series of study sets tailored to PCa and graphically depicted in Fig. 3A. First, we conducted a RPPA experiment on the restricted set of patients referred to as ‘pivotal cohort’, composed only of PCa patients and healthy individuals (HD) (Fig. 3A). Surprisingly, we found that analysis of serum-derived EVs using as low as thirty-seven proteins, selected by their relevance in oncological processes (Fig. S4D), is per se sufficient to infer sample origin (Fig. 3B and Fig. S5A). Hierarchical clustering of RPPA data confirmed that such EV-based proteomic analysis differentiates PCa from HD (Fig. 3C). In particular, ERG (ETS-related gene), PD-L1, Survivin, Integrin-β5, IL-6 and SPARC were significantly altered in tumoral EVs (Fig. 3D). Prospectively, RPPA analysis of key EV (phospho-)proteins such as those involved in the EGFR, mechanistic target of rapamycin (mTOR) and vascular endothelial growth factor receptor (VEGFR) pathways, allow cancer- and patient-specific adoption of treatment strategies (Fig. 3E, F).

It is worth noting that ERG protein over-expression is under investigation as surveyor genomic aberration for the application of novel diagnostic tools in PCa [49]. Along similar lines, PD-L1, Survivin and Integrin-β5 have already been found in vesicles isolated from cancer patients [48–51], SPARC, STAT3 and activated EGFR (EGFR_pY1173), have been described as predictors of response to targeted therapy [52–54] while IL-6 is known to promote prostate tumorigenesis and progression to aggressiveness [52, 55]. Indeed, soluble serum protein components such as IL-6 may eventually contaminate the EV cargo following the differential ultracentrifugation steps [56]. Therefore, we devised an ad hoc experimental design and analyzed a cytokine panel by Luminex technology on two paired samples obtained from the same preparation of serum-derived tumoral EVs. In details, we collected 100 μg of EVs left un-lysed in the supernatant from the last-ultracentrifugation step (SN), likely containing contaminant proteins, along with an identical total protein content (100 μg) from EVs lysed in RIPA buffer (lysed- RIPA) and likely containing residual, membrane-bound contaminants as well as proteins from the EV cargo. While other cytokines were still present in the both samples, IL-6 resulted higher in the RIPA-lysed preparation (Fig. S4E, F). These results suggest that, although soluble serum proteins may still be an undesired contaminant of the EV preparation by differential ultracentrifugation, in our hands the IL-6 levels measured by RPPA are likely to derive mainly from the EV cargo.

In search of further confirmation on the importance of the differentially expressed targets emerging from our RPPA analysis, we evaluated the levels of mRNAs corresponding to selected RPPA antigens (Fig. 3D) in publicly available PCa datasets (https://www.cbioportal.org/ [21] and http://gepia.cancer-pku.cn/index.html [57]). Although tissue mRNA expression represents an analytical and molecular scenario potentially different from released EVs, still we found that half of our RPPA PCa markers were upregulated at the mRNA level when comparing tumor tissues with their normal adjacent counterpart (Fig. S5B, C). Overall our results and, although partial, such additional *in silico* data, strengthen the idea of integrating the available molecular profiling with proteomic analysis of EVs for identifying tumor-specific marker and druggable targets for improvement of patient’s outcome.

### Evaluation of EV protein cargo as a diagnostic tool in PCa

**Fig. 4:**
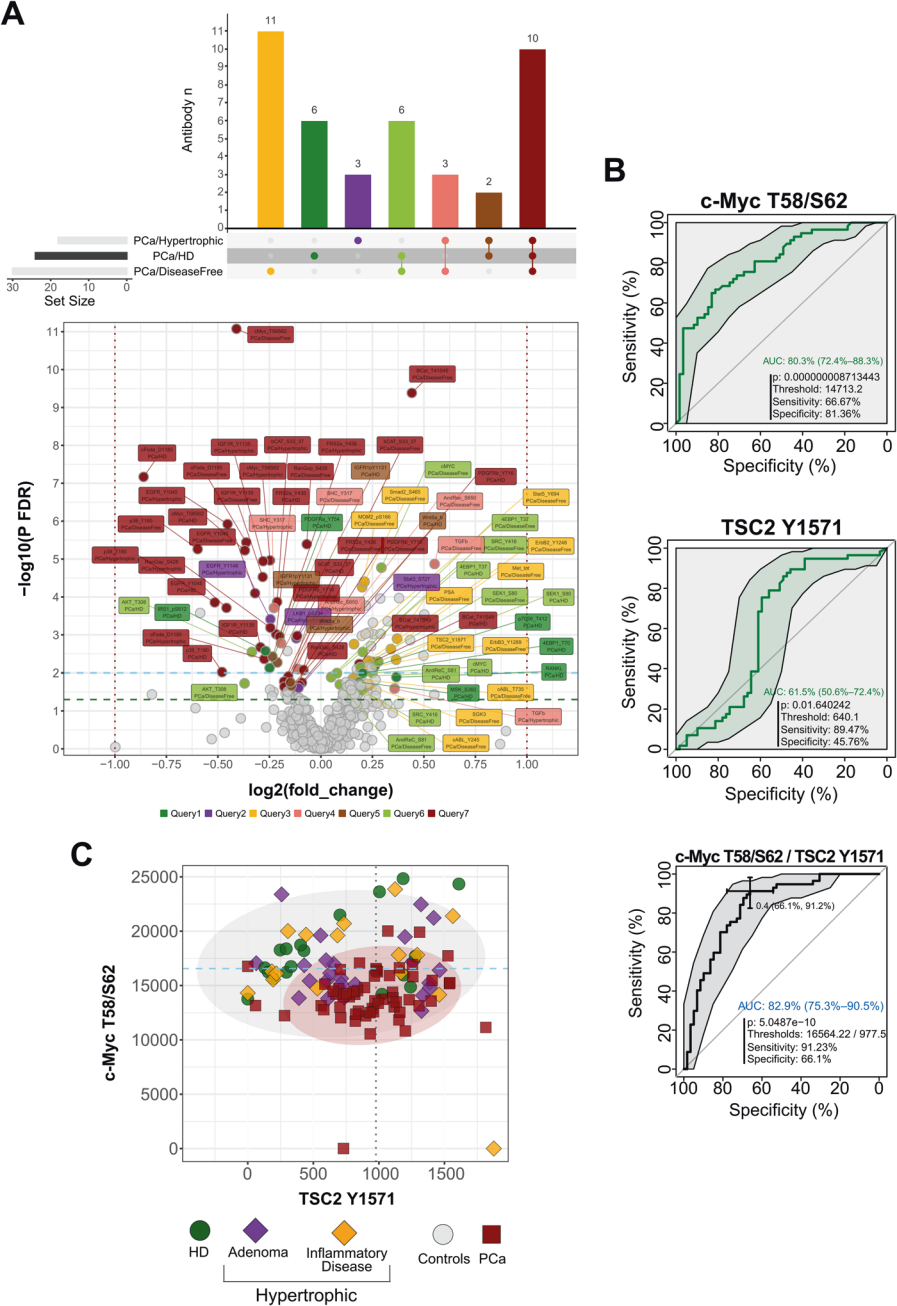
Group comparisons and biomarker analysis on the training cohort. **A** Combined Up Set [94] and volcano plots, with color-coded annotations of diverse RPPA endpoint sets that characterize specific binary comparisons of sample groups. The vertical histogram (upper panel) reports the relative frequency of unique (single dots) and shared (connected dots) significant RPPA antibodies resulting from three specific sets of statistical comparisons, namely PCa versus Hypertrofic, HD and Post-prostatectomy disease free (DF), respectively. The volcano plot in the lower panel shows fold-changes (log2) versus significance [−log10(*p* - value)] for all analyzed comparison sets (i.e. PCa versus Hypertrofic or HD or disease-free, respectively). The color-coding (‘queries’) of RPPA antibody labels in the volcano plot matches the corresponding colors sets in the main frequency histogram. The horizontal histogram adjacent to comparison sets (left-bottom part of the upper panel) shows the absolute frequency of statistically significant RPPA antibodies obtained for each individual set. **B** Univariate ROC curve analysis of two selected, significant candidate markers resulting from statistical comparison of controls (grouped HD plus Hypertrophic diseases) and tumors (upper plots). The diagnostic performance of combined top-scoring candidates was further assessed by ROC curve analysis (bottom plot). All plots report the AUC value along with the 95% confidence interval as well as p, optimal cut-off, sensitivity and specificity values. **C** Bivariate plot of best candidates (c-Myc T58/S62 and TSC2 Y1571) normalized RPPA intensity values in the analyzed cohort comprising tumors (PCa) and control samples, i.e. HD and Hypertrophic diseases, the latter subdivided into Adenoma and Inflammatory diseases, being the category ‘others’ referred to grouped non-neoplastic samples (controls). The ellipses represent the probability of distribution (95% confidence under normality assumption) for the two main comparison groups, namely PCa and others.

In order to expand upon and confirm our preliminary experimental evidence on the pivotal cohort, we analyzed a larger patient set, hereafter referred to as the “training cohort” (Fig. 3A) and composed of: (i) healthy donors (HD); ii) over twelve months disease-free patients (DF); iii) hypertrophic cases (Hyper) and iv) tumors (PCa) (Table S1). Following enrichment by differential ultracentrifugation, EVs isolated from PCa sera showed higher total protein content if compared to HD and DF groups while Hyper showed levels close to those of PCa patients (Fig. S5D). The panel of RPPA endpoints analyzed in the training was enlarged with respect to the pivotal cohort and comprised total and phosphorylated antigens involved in diverse hallmark cancer signaling pathways (Fig. S6). To better define key disease-specific markers, we compared PCa to each of the other groups (i.e. either Hyper or DF or HD) and found significantly different antigens exclusive of individual comparison sets as well as those shared between all the aforementioned comparisons (Fig. 4A, Tables S2–4). Subsequently, we searched for diagnostic candidates based on their individual performance in the ROC curve analysis of PCa versus controls (grouped HD and Hyper). Intriguingly, phospho-c-Myc_T58/S62 and phospho-TSC2_Y1571 emerged as the best candidates and, although their combination resulted in a modest gain of diagnostic power, it might still be of clinical relevance in the context of PCa (Fig. 4B). Graphical representation of the data showed that, compared to controls, at least half of the PCa had lower phospho-c-Myc_T58/S62 combined to higher phospho-TSC2_Y1571 (Fig. 4C). Notably, these results held true even when comparing PCa to the HD group alone (Fig. S7A). Among key endpoints reaching statistical significance in all comparison sets (i.e. PCa versus either Hyper or DF or HD), we found components and interactors of the MAP-kinase/mTOR pathway [58] such as TGF-β, phospho–β-Catenin_T41/S45 (β-Catenin_pT41/pS45) and phospho–c-Myc_T58/S62 itself (Fig. 4A and Tables S2–4, gray boxes), eventually underlying a common aberrant pathway route (Fig. S7B). In addition, several RPPA endpoints emerged as significantly altered or shared when including all available paired comparison sets (i.e. each group versus each of the others) (Fig. S7C).

Interestingly, for a subset of the proteins analyzed in the training, we reproduced the differential expression pattern obtained in the pivotal cohort. In detail, high levels of SPARC were significantly associated with PCas while an increase in PD-L1 and Survivin characterized both inflammatory and neoplastic disease (Fig. S7D). Although ERG did not reach statistical significance in the training cohort, a subgroup of PCa showed elevated (above the 65th percentile) ERG expression and likely associated with high-risk patients as well as with evidence of progression within 3 years (Fig. S8A). Conversely, while in the pivotal cohort IL-6 emerged as a differentially expressed antigen (PCa versus HD), in the training cohort the levels of IL-6 showed a modest, not significant trend of correlation with increasing pathological Tumor-Node-Metastasis (pTNM) staging (Fig. S8B). Intrigued by this result, we sought to correlate key proteomic EV markers with standard clinical parameters such as prostate-specific antigen (PSA) levels and pTNM. While PSA levels in PCa and benign prostatic hyperplasia largely overlap at a range of 4–10 ng/ml [59], in such a gray zone we found, for a few of the analyzed markers, that a fraction of PCa samples was distinguishable from hypertrophic diseases in terms of RPPA expression levels (Fig. S8C, D). Furthermore, a restricted number of RPPA endpoints displayed a correlation trend with pTNM as well as with pTNM and PSA (Fig. S8E).

Overall, the RPPA analysis of EV protein content revealed a set of cancer-specific biomarkers and demonstrated that a targeted liquid-biopsy approach allows discrimination of patients from healthy individuals. Our data suggest that combination of proteomic analysis of EVs with conventional clinical parameters may prove useful for early cancer detection.

### Targeted analysis of EVs proteome as prognostic and monitoring tool in PCa

**Fig. 5:**
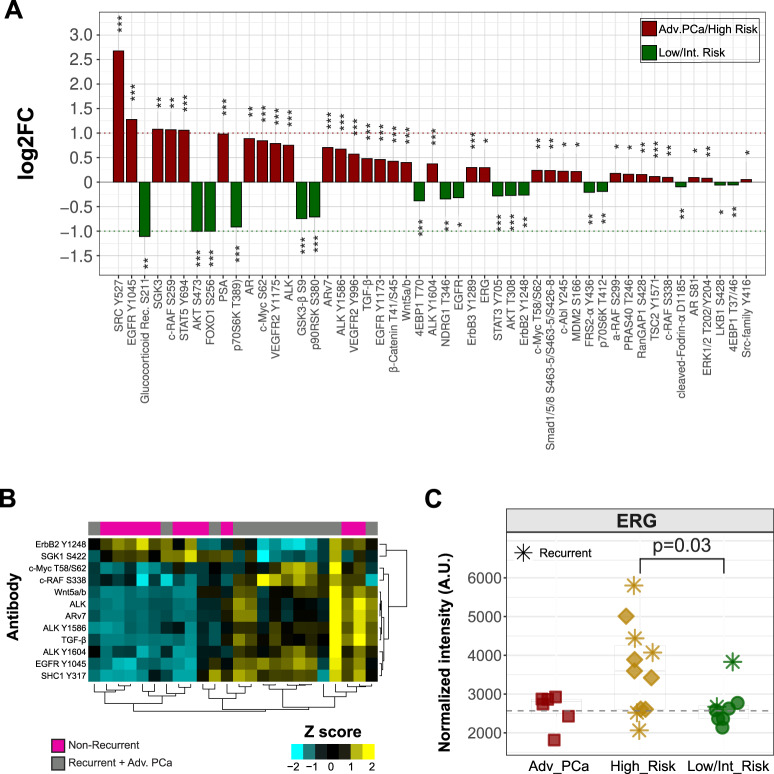
Analysis of EV-based prognostic RPPA biomarkers. **A** Bar chart of log2 fold-change (log2FC) of grouped high-risk and advanced PCa (red) versus low-/intermediate-risk (green) patients for all significantly different RPPA endpoints analyzed by Wilcoxon rank-sum test. Statistical significance reported for each bar is coded with asterisk(s) based on the level of significance (*p < = 0.05, **p < = 0.01, ***p < = 0.001). **B** Two-way unsupervised hierarchical clustering of the 13 significantly different (*p* < = 0.05) RPPA endpoints emerging from statistical comparison of non-recurrent (pink, *n* = 11) and grouped recurrent and advanced tumor (gray, *n* = 13) EV samples. One sample was excluded due to lacking follow-up information. **C** Scatterplots of ERG expression resulting from comparison of low/intermediate-, high-risk and advanced (Adv. PCa) tumors by RPPA analysis. Recurrent patients were indicated by an asterisk (*). The plots represent distribution of RPPA intensity values, and the line indicates an arbitrary baseline. Statistical comparisons were performed as described in the methods section.

Due to the relatively high life expectancy of diagnosed patients, the PCa risk management is a fundamental research field [60]. To identify biomarkers that are predictive of the risk of progression, we studied a retrospective cohort (Risk Assessment, Fig. 3A, Table S5) comprehensive of six advanced castration-resistant (CR) patients as reference controls. Serum samples were collected before surgery for the 19 primary tumor cases and during the recurrence phase for the advanced CR patients. Isolated EVs underwent RPPA profiling and, interestingly, high-risk and advanced tumors showed shared expression of several antigens (Fig. S9A). Therefore, in order to find a common signature, we grouped high-risk patients with advanced tumors and compared them to low/intermediate-risk cases (Fig. 5A). Moreover, we compared high-risk patients alone to either advanced PCa or low/intermediate-risk cases (Fig. S9B, C). The differentially expressed antigens characterizing the high-risk and advanced PCa patients and comprising components of the cell cycle, PI3K, WNT, and MAPK pathways, likely reflect the higher frequency of genomic alterations found in aggressive forms [21]. Indeed, mining of publicly available PCa tissue dataset [21] confirmed an increased number of mutations in advanced forms, both at the pathway and at the gene level, if looking at the genes corresponding to the differentially expressed RPPA endpoints (Fig. S9D, E). Then, we investigated the association between RPPA antigen levels and the frequency of recurrence in the retrospective, i.e. Risk Assessment, cohort (Fig. 3A). Seven out of 19 primary cases–with the exclusion of one sample lacking a documented follow-up–developed metastases over a 15-year time frame (Table S5). Intriguingly, heatmap with significantly different RPPA endpoints showed that the non-recurrent cases converged into a separate cluster when compared to grouped recurrent cases and advanced tumors (Fig. 5B), advocating for potential prognostic implications of such an analytical approach. Along these lines and similar to the strategy used to individuate the best diagnostic candidates in the training cohort, we performed statistical comparisons and ROC curves and found RPPA targets that may allow for EV-based discrimination of recurrent cases and advanced forms. In particular, phospho-c-Myc_T58/S62, phospho-SHC_Y317 and Wnt5a/b as well as phospho–c-RAF_S338 and TGF-β reached statistical significance and emerged as optimal prognostic candidates (Fig. S10A and Fig. S10C, individual antigens). Advanced PCa has the propensity to metastasize to bone and, notably, TGF-β mRNA over-expression is associated with tumor invasion and spreading to bones [61]. Therefore, we investigated a publicly available dataset (GEO GSE74685 [61]) and found that, consistently with our results obtained in EVs (Fig. S10C), the levels of tissue mRNAs for genes corresponding to RPPA candidates, i.e. c-Myc, SHC-1, Wnt5a, c-RAF-1 as well as TGF-β, were increased in bone metastases as compared to all other metastatic sites (Fig. S10D). We then asked whether any combination of these five antigens could gain prognostic and predictive value over individual ROC curves. Of note, the concomitant random expression of almost three out of the five candidate RPPA endpoints at levels beyond their respective cut-offs, was able to produce a significantly improved prognostic signature (Fig. S10C, Score). Again, we sought to corroborate our results by analysis of mRNA levels of the corresponding genes from publicly available gene expression datasets, namely [21] and [62]. When comparing metastatic and primary PCa samples, only Wnt5a/b was significantly and congruently upregulated in both datasets (Fig. S10E), suggesting that our data and EV-based approach provide complementary information to standard tissue transcriptomic characterization. Interestingly and similarly to what we have shown here for the pivotal and training sets and others have already shown [63, 64], we observed ERG protein expression beyond the calculated cut-off (i.e. over-expressed) in high-risk patients (Fig. 5A) and in low- and high-risk categories (Fig. 5C). Of note, elevated ERG protein expression in recurrent patients (Fig. 5A) mirrored gene expression data from two public datasets (GEO GSE32269 [62] and GSE74685 [61], Fig. S10F, G, respectively), whereby ERG over-expression has been shown to parallel the genomic fusion and to contribute to PCa progression by WNT and MAPK signaling activation [61].

Finally, to confirm the prognostic relevance of the data, we took advantage of gene expression profiling (Affymetrix arrays) on the ex vivo collection of primary PCa cultures (*n* = 40, Prognostic Marker Evaluation, Fig. 3A) from patients with 10-year documented clinical follow-up [65, 66]. Gene panels enriched in primary cells derived from recurrent and non-recurrent cases were assigned either bad or good prognosis, respectively (Fig. S11A). Surprisingly and in line with our RPPA results, cells obtained from patients with poor prognosis displayed mRNA upregulation for the genes corresponding to significant prognostic RPPA candidates, i.e. TGFΒ1, WNT5A/B, SHC1, RAF1 and MYC (Fig. S11A, B). Western blotting analysis confirmed the over-expression of candidates in cells deriving from cases with poor prognosis (Fig. S11C). Overall, these data corroborate the hypothesis that an EV-based protein signature may have a clinically valuable prognostic significance.

## Discussion

Shreds of evidence have shown that EVs are an important source of cancer biomarkers [67]. Since for many cancers the amount of vesicles circulating in patients’ biological fluids is acceptedly increased as compared to healthy individuals, the analysis of EV proteomic patterns [25] may provide a valuable strategy to complement current liquid-biopsy technologies, such as circulating tumor cells or DNA (ctDNA) analysis.

Here, we used the RPPA platform for multiplexed, high-throughput analysis of protein cargo in cancer-derived EVs. We first tested the sensitivity and specificity of this approach for the detection of selected proteins in EVs isolated from commercially available cancer cell lines. In particular, EpCAM expression was analyzed in EVs by RPPA assay and furtherly validated by immunogold electron microscopy and Western blotting.

Commercially available ELISA assays for EVs are based on EV immune-capture or lysate analysis [27, 68]. Here, we devised a new homemade ELISA assay, i.e. ELEXO, as both an additional validation platform and a potential alternative to RPPA for rapid translation to the clinics of a low-throughput quantification assay for surface EV antigens.

To date, blood PSA screening is the sole non-invasive test for PCa diagnosis. In addition, among patients with elevated serum PSA levels (≥3–4 ng/mL) and undergoing a standard transrectal ultrasound-guided biopsy, the chance of detecting PCa is approximately 30–40% [69]. Consequently, there is an urgent need for fast and multiparametric diagnostic tests based on non-invasive methods and requiring low amounts of patients’ sample. In this regard, we conceived a pilot study on EVs isolated from a small cohort of PCas and found that a proteomic repertoire of as low as 37 antigens allows for the discrimination of tumors from healthy individuals. Interestingly, among the differentially expressed antigens found by us, IL-6 has already been described as a tumor-discriminating antigen in PCa [70], while high levels of PD-L1, Integrin-β5 and Survivin upregulation seems to correlate with cancer survival and aggressiveness [50, 51, 71, 72]. Subsequently, we extended the proteomic analysis to EVs isolated from a larger PCa cohort (training) including healthy donors and hypertrophic patients as control groups as well as post-prostatectomy disease-free patients as an additional comparative group. Again, based on the sole analysis of EV content, we found a proteomic signature capable of distinguishing tumors from controls. Of note, the set of differentially expressed endpoints include key players in cancer signaling networks. In particular, phosphorylation of β-catenin at T41-S45 determines its nuclear, trans-activating activity [73], TSC2 phosphorylation (Y1571) associates with mTOR signaling activation [58, 74] and, intriguingly, the latter is coupled to low phospho-c-Myc_T58/S62, i.e. reduced c-Myc degradation rates [75, 76]. The cartoon depicted in Fig. S7B exemplifies an hypothetical aberrant network flow, whereby i) an increased inhibitory phosphorylation of phospho–TSC2_Y1571 activates mTORC1 signaling, ii) augmented phospho-c-Abl_T735, directly relating to its sequestration into the cytoplasm by 14–3–3 proteins, promotes TGF-β activity [77] and iii) SEK1 phosphorylation favors metastatic spreading.

While pTNM and Gleason score are critical parameters to establish the risk of recurrence [78], their prognostic value remains poor leaving a clinical gap in the identification of PCas that have an intrinsic tendency to progress. Therefore, we studied a retrospective cohort, with 15 years of documented follow-up, to identify EV proteins indicative of tumor aggressiveness. Patients from this cohort were stratified into low/intermediate- and high-risk cases and were analyzed together with a group of advanced tumors. Intriguingly, ERG over-expression correlated with advanced PCa forms and high-risk cases as well as with recurrence. High-risk and advanced PCas shared the expression of diverse proteins that are reportedly associated with cancer progression, namely TGF-β [79], Wnt5a/b [34, 35, 80], Shc1 (phospho-SHCY317) [28], phospho-c-RAF-S338 and phospho-c-MycT58/S62 [81]. Moreover, we validated our results by measuring the expression of corresponding candidate genes on a collection of patient-derived immortalized cells [66] and confirmed the correlation between increased mRNA levels and a documented poor prognosis.

Since the management of PCa patients is currently under heated debate [82, 83], our predictive molecular signature may provide the opportunity to discriminate risk categories, improve the accuracy and feasibility of early diagnosis and suggest a potential monitoring method for suitable assignment of active surveillance protocols, nerve-sparing conservative surgery as well as radical treatments.

Although our data deserve further validation in dedicated, independent studies, the integration of the proteomic analysis of EVs with other available clinical and molecular parameters may lead to next-generation, multiparametric liquid-biopsy assays, thus providing a novel tool for screening, diagnosis, and risk assessment of PCa as well as of other cancers.

## Materials and methods

### Patient recruitment

The study design is outlined in Fig. 3A and clinical data were reported in Table S1. More specifically, a pivotal cohort composed of 12 primary prostate tumors and 16 healthy donors were used as technical proof of principle. The training cohort was composed of (i) 57 diagnosed primary tumors with 3-year clinically documented follow-up, (ii) 41 hypertrophic patients, (iii) 44 cases enrolled at least 12 months follow-up after prostatectomy and negative for recurrence or residual disease, and (iv) 18 age-matched healthy donors. A retrospective, risk assessment cohort (15-year follow-up; Table S5) including 19 tumor patients for prognostic marker estimation as well as 7 recurrent and 11 non-recurrent patients (and one additional patient with non-documented follow-up).

PCa patient stratification was performed by considering D’Amico risk categories [84], and ISUP/WHO 2016 classification, but arbitrarily stratifying cohorts as follows: *Low risk:* Gleason =6 and pTNM ≤ T2a; *Low/Intermediate risk:* Gleason ≤ 7(3 + 4) and pTNM = T2b-2c; *High risk:* Gleason = 7(4 + 3) and pTNM = T2c and all Gleason score cases with pTNM ≥ T3a. Cohorts were enrolled before COVID-19 pandemic. [pTNM: pathological Tumor-Node-Metastasis (pTNM). The NSC-lung cancer and colorectal tumor cohort clinical data were reported in Table S1.

### EV separation

EV isolation was optimized as previously described [28] and following the MISEV2018 guidelines [30] Cell lines were cultivated in recommended medium and FBS (Fetal bovine serum) starved before EV concentration and processed with round of centrifugation as follows. Cell conditioned-starved medium was centrifuged 5 minutes at 1200 rpm after 72 h. The supernatants transferred in new tubes and centrifuged again for 15 minutes at 13300 rpm. Supernatants were transferred in clean tubes and ultracentrifuged (Beckman Coulter, Brea, CA) 2 h at 110,000*g* (RCF). All centrifugation steps were performed at 4 °C temperature. (Beckman Coulter Optima-LE-80K-Ultracentrifuge; Rotor: BECKMAN SW41). Pellets were washed once and resuspended in 40 μl of phosphate buffer saline (PBS). The concentration of EV suspension was measured by Bradford assay. 5 ml of peripheral blood from participants were collected in serum clinical tubes (Code-367955-DBdiagnostics). Sera were collected and centrifuged 5 minutes at 1200 rpm for serum separation and stored at −80 °C. 1 ml of serum for patient was used to obtain about 100–300 total μg of EVs. General patient exclusion criteria: patients with blood viral infection and different kind of tumors. For EVs separation, sera were thawed, transferred in clean tubes and diluted in PBS to reach equal volume of 1.4 ml. They were centrifuged 5 minutes at 1200 rpm, the supernatants transferred in new tubes and centrifuged again for 15 minutes at 13,300 rpm. Supernatants were transferred in clean tubes and ultracentrifuged (Beckman Coulter, Brea, CA) 2 h at 160,000*g* (RCF). All centrifugation steps were performed at 4 °C temperature. (Sorvall-Ultracentrifige WX90-Ultra series, SN 42071706, ASHI; Rotor F50L-24×1.5). Pellets were washed once and resuspended in 40 μl of phosphate buffer saline (PBS). The concentration of EV suspension was measured by Bradford assay.

### Reverse-phase Protein microArrays

Reverse-Phase Protein microArrays (RPPA) were already optimized [28, 85]. Briefly, all PBS-resuspended EV samples were lysed in a TPER (Thermo Fisher Scientific)-based lysis buffer for 30 minutes on ice. EV lysates were properly diluted and then printed in triplicate spots on nitrocellulose-coated glass slides (GRACE Bio-Labs, Bend, OR) using an Aushon 2470 equipped with 185 μm pins (Aushon Biosystems, Billerica, MA), according to the manufacturer’s instructions at a concentration of 0.5 mg/ml and 0.125 mg/ml. Reference standard lysates, comprised of HeLa + Pervanadate (BD, Franklin Lakes, NJ), Jurkat + Etoposide (Cell Signaling, Danvers, MA), Jurkat + Calyculin A (Cell Signaling), A431 + Pervanadate (Santa Cruz Biotechnologies) and A431 + EGF (BD, Franklin Lakes, NJ) were printed in 10-point dilution curves as procedural controls and positive controls for antibody staining. Each reference was printed in triplicate at concentrations of 0.5 mg/ml and 0.125 mg/ml. Per-spot protein concentration was evaluated by Sypro Ruby Protein Blot Stain (Invitrogen, Carlsbad, CA). Immediately prior to antibody staining, printed slides were treated with 1x ReBlot Mild Solution (Merck Millipore, Darmstadt, Germany) for 15 min, washed 2 × 5 min with PBS (Invitrogen) and incubated for 2 h in blocking solution (2% I-Block, (Applied Biosystems, Foster City, CA), 0.1% Tween-20 in PBS). Immunostaining was carried out using an avidin-biotin complex (ABC) signal amplification kit (DAKO, Carpinteria, CA). Primary antibody binding was detected using a biotinylated goat anti-rabbit IgG H + L (1:7500) (Vector Laboratories, Burlingame, CA) or rabbit anti-mouse IgG (1:10) (DAKO) followed by biotin amplification and streptavidin-conjugated IRDye680LT fluorophore (LI-COR Biosciences, Lincoln, NE). Primary antibodies against total and phosphorylated protein targets, were previously validated for single-band specificity by Western Blot using cell lysates (see Supplementary File 1). Negative control slides were incubated with secondary antibody alone. All Sypro and immunostained slides were scanned using a Tecan Power Scanner™ (Tecan Group Ltd, Switzerland). Acquired images were analyzed by MicroVigene v5.2 (VigeneTech, Carlisle, MA) software for spot detection, local background subtraction, negative control subtraction, replicate averaging, and total protein normalization. Throughout the manuscript and in the figures, the above-described normalized RPPA data are referred to as normalized RPPA intensity or levels and are expressed in arbitrary units (A.U.).

### Electron microscopy

For Scanning Electron Microscopy (SEM) analysis, purified EVs were left to adhere to polylysine treated round glass coverslips (10 mm) for 4 h at RT and then fixed with glutaraldehyde 2,5% in sodium cacodylate buffer 0.1 M overnight at 4 °C. Samples were washed, post-fixed with 1% OsO4 in 0.1 M sodium cacodylate buffer for 1 h at RT and dehydrated through a graded series of ethanol solutions (from 30% to 100%). Ethanol was gradually substituted by a 1:1 solution of hexamethyldisilazane (HMDS) and absolute ethanol for 30 min, successively by pure HMDS for 1 h (RT). Samples were completely dried by removing the HMDS and leaving at RT for 2 h. Dried samples were mounted on stubs, coated with gold (20 nm) and analyzed in a FE-SEM Quanta Inspect F (FEI, Thermo Fisher Scientific). (Shively and Miller, 2009).

For Transmission Electron Microscopy (TEM) purified EVs from cell-conditioned supernatants or serum were analyzed by negative staining method according to [86, 87] with slight modifications. Briefly, samples were let to adsorb on 400mesh carbon-coated grids for 10 min, blotted by filter paper and air dried. An ammonium molybdate 4% and phosphotungstic acid 2% (50% v/v) contrasting solution (pH 6.8) was added for 20 s to each sample and blotted by filter paper. Samples were examined at 100 kV by Philips EM208S TEM (FEI - Thermo Fisher Scientific), equipped with the Megaview II SIS camera (Olympus).

EV TEM images were analyzed by iTEM software to collect the diameter size of more than 100 vesicles for each sample. Only vesicles more than 20 nm in diameter were taken into account and the EV size distributions were expressed in a box plot.

Immuno-Electron Microscopy (IEM) was performed on extracellular vesicles adsorbed on carbon coated grids prepared as previously described for TEM negative staining. Grids were floated side down on a drop of PBS buffer and successively transferred on a drop of anti-EpCAM monoclonal antibody (Santa Cruz) (1:5 in PBS) over night at 4 °C. Then, samples were rinsed on PBS drops and incubated on 10nm gold-conjugated goat anti-mouse IgG serum (SIGMA) (1:30) for another 2h. Finally they were rinsed and stained by an ammonium molybdate 4% (pH 6,8) and phpsphotungstic acid 2% solution (50% v/v). The excess fluid was blotted by filter paper and grids were air dried and observed Philips EM208S TEM (EI –ThermoFisher).

### Cytofluorimetric and Western blot assay

#### Flow cytometric analysis

Cancer cell lines (100,000 cells/tube) were incubated with antibodies against EpCAM (Miltenyi, Biotec, Custom antibody) and PD-L1 (XP version, Cell Signaling Danvers, MA) for 1 h on ice. Anti-IgG1 antibody was used as negative control (Abcam, Cambridge, UK). After 3 washes in PBS, samples were incubated with PE- or FITC-conjugated secondary antibodies for 1 h on ice. FACS analysis was performed by FACS-Canto instrument (BD, Becton-Dickinson, Franklin Lakes, NJ, USA).

#### Western blotting

EVs were resuspended in RIPA lysis buffer and incubated for 15 minutes in ice. TPER (Thermo Fisher Scientific)-based lysis was used for sera EVs. Samples were centrifuged at 16,000 x g and supernatants were collected and transferred in a clean tube. After Bradford assay, EV lysates were diluted in LDS sample buffer (Thermofisher Scientific, Massachusetts, USA) at total amount of 40μg. Samples were loaded and run into a 4–12% polyacrylamide gel (Thermofisher Scientific, Massachusetts, USA) and transferred on nitrocellulose filters (SDS-PAGE) that were subsequently incubated with anti-EpCAM and anti-CD81 (Santa Cruz Biotechnology, California, USA) antibodies at 4 °C overnight. TSG101 and CD81 antibodies were used following relative datasheets Abcam and Santa Cruz. Then, filters were incubated with HRP-conjugated secondary anti-mouse or rabbit antibody (Southern Biotech, Birmingham, USA) for 1 h and signals detected with ECL method (GE Healthcare, Piscataway Township, NJ) by Chemidoc XRS system (Bio-Rad, Hercules, CA).

### Homemade ELISA ‘ELEXO’

EVs were resuspended in PBS (50μl) and deposited in a 96well-plate (Nunc, Milan, IT) at final concentration of 10 µg/well and incubated for coating for 2 h at 37 °C (or over-night). After the coating, three washes in PBS were performed. Coated plate was treated with blocking solution [PBS with 0.5% BSA (Bovine Serum Albumin pure protein); the solution was filtered with 0.200 μm pores] for 2 h. Primary Antibody staining was performed in PBS solution and incubated for 1 h at room temperature while secondary antibody (anti-mouse-HRP-conjugated antibody; Southern Biotech, Birmingham, USA) was diluted in PBS/0.5% BSA solution for 1 h at room temperature. Anti-PD-L1 (86744-Cell Signaling Technology, Danvers, MA), anti-IgG1 (Abcam, Cambridge, UK), anti-CD81 Mab and anti-EpCAM Mab (Miltenyi Biotec, USA; Custom antibody) were used at final concentration of 2 μg/ml. The plate was washed 3 times in PBS after each antibody incubation. After washing, the color-developing reaction with TMB (Thermoscientific, Massachusetts, USA) was started and then stopped with H2SO4. Optical densities were measured at 450 nm by Victor X3 (Perkin Elmer, Massachussets, USA). The blocking solution and the working antibody concentrations were optimized to minimize background staining with a relative scalar dilution of the reagents (i.e. BSA, primary and secondary antibodies).

### Luminex assay

100 μg of EVs were lysed in 50 μl of standard RIPA buffer [(20 mM Tris-HCl pH7.2150 mM NaCl;1% NP40 (Igepal CA-630); Distilled water to volume; Proteases-inhibitors)] and diluted 1:4 in PBS for the analysis. 100 μg of parental EVs were left, non-lysed (SN), in 50 μl in the buffer (PBS) of the last step of Ultracentrifugation and was directly analyzed by Luminex. Cytokine/chemokine quantification in EV extracts and in EV(SN) was achieved by xMAP technology through a Luminex platform (Bio-Rad Laboratories, Hercules, CA, USA) equipped with a magnetic washer workstation according to the manufacturer’s protocol. RIPA (dilute 1:4 in PBS) and PBS buffer were used as background controls. Samples were analyzed using a human magnetic Luminex assay (R&D Systems, Minneapolis, MN, USA). Brain-Derived Neutrophil Factor (BDNF), CCL11, Fibroblast Growth Factor 13 (FGF-13), IL-5, IL-4, IL-23, IL-6, MMP-2 (membrane-matrix-metalloprotease-2), beta-Nerve Growth Factor (beta-NGF), N-regulin-1 beta1/NRG-1, Tumor Necrosis Factor alpha (TNF-α), Interferon gamma-induced protein 10 (CXCL10), Interferon gamma (IFN-γ), IL-2, IL-8/CXCL8, IL-17/IL-17A, CCL-2/MCP-1 and Vascular Endothelial Growth Factor (VEGF) were analyzed. The quantification was carried out with a Bio-Plex array reader (Bio-Plex 200 System) and Bio-Plex Manager (Version 6.1 Bio-Rad Laboratories, Hercules, CA, USA) software.

### Statistical analysis and data representation

#### Protein analyses in EV samples

Student’s t or non-parametric Wilcoxon rank-sum tests were used for continuous variables to analyze the differences between groups. A p-value ≤ 0.05 was considered statistically significant. Furthermore, the receiver operating characteristic (ROC) method was used in order to find possible optimal cut-offs of the biomarkers capable of splitting patients into groups with different outcomes probabilities. Statistical analyses were conducted independently by means of SPSS® (v21.0) and MedCalc® (v10.0.1) or ‘R’ [88]. Data standardization (scaling), followed by two-way hierarchical clustering (Euclidean distance and Ward’s method was used if not specified elsewhere), were performed by means of JMP v11 (SAS Institute, Cary, NC) or ‘R’ [88] and RStudio [89]. Principal component analysis (PCA) as well as most data represented throughout the manuscript was independently reproduced by means of ‘R’ using the following packages: base, methods, utils, stats, graphics, grDevices, tcltk, openxlsx, tidyverse [90], data.table, RColorBrewer, reshape2, reshape, readxl, FactoMineR, factoextra, grid, gridExtra, circlize, cluster, dendextend and ComplexHeatmap [91].

#### Analysis of publicly available datasets

Messenger RNA results from Taylor’s tissue dataset (NCBI GEO accession code GSE21032) have been accessed through the Prostate Cancer Genomics Data Portal (http://cbioportal/) and combined with reported clinical data. Wilcoxon/Kruskal Wallis was used to analyze the differences between groups. GraphPad Prism v4 and JMP v11 (SAS Institute, Cary, NC) were used to perform statistical analyses.

### Cell lines, PCa-derived cells and gene expression profiling (Affymetrix)

#### Cell cultures

All cell lines were obtained by ATCC. All cells were used as precocious (six passages) frozen stocks after arrival. They are routinely tested for Mycoplasma contamination (“PCR mycoplasma test kit”, product no. A3744, PanReac AppliChem) before EV preparations. H1299, HT1975, HT29 cells were cultivated as recommended protocols. SW480 line was maintained in RPMI and 10% of Fetal Bovine Serum (FBS). A431 and 293T cells were cultivated in DMEM (Dulbecco’s Modified Eagle’s Medium) with 10% of FBS, Glutamine (Gln) and Penicillin–Streptomycin (P/S) at standard doses. 293T cells were stable transduced with TWEEN vector [92] empty (Control) or with PD-L1 gene. Sequence-verified cDNA encoding for human PD-L1 was purchased from Dharmacon, cut with XbaI and EcoRV and inserted into TWEEN vector. It was kindly provided by Dr.Valeria Coppola. (PD-L1 Human-MGC Human CD274 Sequence-Verified cDNA-Clone ID: 30915301-Catalog Number: MHS6278-202856825)

(Lentiviral manipulation authorized by Ministry of Health rules. RM/IC/Op2/17/002.notifica I.5.i.s/2017/15 - Biotecnologie. D.L.vo 206/2001). PC20 cancer activated fibroblast were obtained by tumor primary tissue cultures as by previously published [93].

#### PCa-derived cells

Primary PCa cultures were derived from freshly-explanted tissue specimens (PCa-derived ex vivo model) following immortalization and phenotypic characterization. Clinical data and outcome of patients were collected for 15 years [66]. Briefly, poor prognosis group of donor patients with clinically localized PCa was defined by the presence of biochemical/local recurrence, metastasis, or disease-specific mortality, while the good prognosis group was defined by complete remission after surgery alone. Prognostic signature “Bad versus Good Prognosis profiling” was obtained in PCa cells by Affymetrix array (Human U133A Gene ChIP platform) using PCa cells derived from patients with different progression of disease (recurrent versus non-recurrent disease). Regulated biological processes were identified by the GOAL Web-based application and Gene Ontology (GO) terms with *p* < 0.01 considered differentially regulated (false discovery rate = 0.013).

#### Affymetrix data analysis

Affymetrix Gene Chip scanning was analyzed by customized R language-based script [88] using Bioconductor (http://www.bioconductor.org) for quality-control analysis, data normalization, hierarchical clustering, and identification of differentially expressed transcripts. Biological processes and molecular functions involved were identified by the GOAL Web-based application and the Unigene Build 154 according to the Gene Ontology (GO; http://www.geneontology.org) Consortium classification. Genes reported with *p* < 0.01 were considered differentially regulated (false discovery rate = 0.013 [65, 66]).

## Supplementary information

Supplementary Tables (S1-S5)

Supplementary Figure and Table Legends

Supplementary Fig.S1

Supplementary Fig.S2

Supplementary Fig.S3

Supplementary Fig.S4

Supplementary Fig.S5

Supplementary Fig.S6

Supplementary Fig.S7

Supplementary Fig.S8

Supplementary Fig.S9

Supplementary Fig.S10

Supplementary Fig.S11

Supplementary Material
