# Supplementary material for: Diagnostic and prognostic potential of the proteomic profiling of serum-derived extracellular vesicles in prostate cancer
Source: Cell Death Dis. 2021 Jun 21;12(7):636. doi: 10.1038/s41419-021-03909-z (PMC8215487; doi:10.1038/s41419-021-03909-z)
Supplement: Supplementary file 2 — Supplementary Figure and Table Legends [file 41419_2021_3909_MOESM2_ESM.docx]

**Supplementary Figure Legends and Tables**

**(Legends for Figures S1 to S11 and Tables S1-5)**

**Fig.S1**

*A)* Schematic representation of the steps of EV separation by differential ultracentrifugation *B)* RPPA analysis of protein associated with EVs purified from several cell lines. Data were representative of triplicates and reported with SD. *C)* Representative images of Western blotting of the endogenous protein TSG101 and CD81 in H1975 and H1299 EVs. *D*) H1975 and H1299 cells, positive and negative for EpCAM expression respectively, analyzed by Cytofluorimetric assay. IgG isotype was used as internal control. Representative images were reported. *E*) Western blotting of EpCAM in EVs isolated from H1975 (EpCAM^+^) and H1299 (EpCAM^-^) cell lines. CD81 expression was used as endogenous control. A representative image was reported. *F*) EpCAM expression evaluated in H1975- and H1299-derived EVs by RPPA assay. EVs isolated from H1975 and H1299 cell cultures, were mixed at varying percentages, following a reciprocal dilution curve. Data are shown as mean and SD (n=3). *G*) Cytofluorimetric analysis of EpCAM in HT29 (EpCAM^+^) cell line. IgG isotype was used as internal control. *H*) EpCAM expression evaluated in HT29- and H1299-derived EVs by RPPA. HT29 (EpCAM^+^) and H1299 (EpCAM^-^) were mixed following a reciprocal dilution curve, reported as relative percentage of HT29/H1299 ratio. Data are reported as mean and SD (n=3).

**Fig.S2**

*A)* Representative images of Western blotting of EpCAM, TGF-β, IL-6 in LNCaP and PC3 cell lines. GAPDH was used as endogenous control. *B*) LNCaP-EV (EpCAM^+^) and PC3-EV (EpCAM^-^) were mixed following a reciprocal dilution curve, reported as relative percentage of LNCaP/PC3 ratio. Data are reported as mean and SD (n=3). *C-D*) LNCaP-EV and PC3-EV were mixed following a selected dilution curve, reported as relative percentage of LNCaP/PC3 ratio, run together with pure cytokine IL-6 and TGF-β and analyzed by RPPA. Data are reported as mean and SD (n=3). *E-F)* RPPA analysis of EGFR and PD-L1 (E) or EGFR_pY1068 (F) antigen levels in EVs isolated from cultures of A431 cells and human fibroblasts. EV samples were first diluted to a total protein concentration of 0.5 μg/ml and then printed as mixtures with the indicated decreasing and increasing fractions of A431 and fibroblasts, respectively. Represented data correspond to mean and SD (n=3) of RPPA intensities obtained after normalization of raw data versus total protein content and subtraction of secondary antibody background.

**Fig.S3**

*A*) Western blotting of EpCAM in HT29- and SW480-derived EVs. CD81 expression was used as endogenous control. A representative image was reported. *B*) EpCAM expression was evaluated in HT29- and SW480-derived EVs by ELEXO assay. IgG isotype and Phosphate-Buffered Saline (PBS) were used as baseline control. CD81 antigen was used as endogenous expression control. Data were reported as the mean and SD (n=3) of arbitrary units of O.D. (Optical Density) at the specified wavelength (nm). *C*) 293T embryonic kidney cells were transduced with lentiviral vector TWEEN expressing PD-L1 (PD-L1) and EVs were isolated from relative conditioned cultures. PD-L1 EVs were analyzed for antigen level by ELEXO assay. EVs obtained from 293T cells transduced with empty vector, TWEEN, were used as negative sample. PD-L1 primary and secondary antibodies in PBS condition (PBS) were used as background control. Data were reported as the mean and SD (n=3) of arbitrary units of O.D. (Optical Density) at the specified wavelength (nm). *D*) PD-L1 antigen expression in H1299 and H1975 cells, analyzed by Cytofluorimetric assay. IgG isotype was used as an internal control. Representative images were reported. *E*) PD-L1 antigen expression in H1299- and H1975-derived EVs, analyzed by ELEXO. Data were reported as the mean and SD (n=3) of arbitrary units of O.D. (Optical Density) at the specified wavelength (nm). Results were reported as normalized over IgG isotype and PBS values. *F*) PD-L1 antigen expression was analyzed by RPPA in whole-cell and corresponding EVs lysates in H1299, H1975, HT29, SW480, A431 cell lines.

**Fig.S4**

*A)* Representative images of TEM of EVs isolated from the sera of prostate cancer patients. Red numbers were representative of vesicle diameters. *B)* Representative images of Western blotting of EVs purified from sera of donors and patients, i.e four HD (healthy donors), Hyper (hypertrophic cases) and PCa. 20 μg of each point were loaded in different running gel (Running Gel 1 and Running Gel-2, representative images) for TSG101 and CD81 protein analyses in the same sample. *C*) RPPA analysis of protein associated with EVs purified from twelve prostate cancer patients of pivotal cohort. Data were reported as mean and SD, (n=3). *D)* Endpoints selected for RPPA analysis of EV samples of the pivotal cohort. *E-F*) Cytokine/chemokine quantification in EV extracts and in post-Ultracentrifugation- buffer (PBS) contained non-lysed EV(SN) by Luminex assay. Brain-Derived Neutrophil Factor (BDNF), CCL11, Fibroblast Growth Factor 13 (FGF-13), IL-5, IL-4, IL-23, MMP-2 (membrane-matrix-metalloprotease-2), beta-Nerve Growth Factor (beta-NGF), N-regulin-1 beta1/NRG-1, Tumor Necrosis Factor alpha (TNF-α), Interferon gamma-induced protein 10 (CXCL10), Interferon gamma (IFN-γ), IL-2, IL-8/CXCL8, IL-17/IL-17A, CCL-2/MCP-1 and Vascular Endothelial Growth Factor (VEGF) and IL-6 (*F*) were analyzed. Data were reported as mean and SD, (n=2).

**Fig.S5**

*A)* Scatterplots of additional RPPA endpoints analyzed in the pivotal cohort and showing statistical significance when comparing PCa- and HD-derived exosome samples. Wilcoxon rank sum test results are coded with asterisk(s) based on the level of significance (* p<=0.05, ** p<=0.01, *** p<=0.001). *B) In silico* analysis of prostate cancer datasets publicly available on cBioportal (<http://www.cbioportal.org>; (1)) mRNA expression of ERG, Survivin (BIRC5), SPARC, Integrin-Beta 5(ITGB5), CD274 (PDL-1) and IL-6, in primary (T=131) and metastasis (M=19) versus (vs) Normal (N=29) tissues as by analysis of published data. Statistical analysis (Wilcoxon/Kruskal-Wallis Test) suggested a non-significant trend for SPARC, PD-L1, Integrin-β5 (ITGB5) and a significant difference for Survivin, ERG and IL-6. (The level of significance: * p<=0.05, ** p<=0.01, *** p<=0.001). *C*) Gene expression levels (RNAseq) of ERG stratified by the presence of ERG fusions and mutations in the TCGA PanCancer Atlas and Taylor’s datasets (source cBioportal and (1)). *D*) Total protein concentration (μg/mL) of EVs isolated from 1 mL of serum of Healthy donors (HD), Disease-free (DS), Hypertrofic (Hyper) and tumour (PCa) samples. The comparison among group were performed by Wilcoxon/Kruskal-Wallis Test.

**Fig.S6**

List of 84 RPPA analytes measured in the study, grouped by key cancer-related pathways.

**Fig.S7**

*A)* Best performing binary combinations of c-Myc T58/S62 in ROC curve analysis of PCa versus HD. All plots report the AUC value along with the 95% confidence interval and the p, optimal cut-off, sensitivity and specificity values. *B*) Schematic representation of signaling cascades for a specific subset of analyzed RPPA endpoints with key, cancer-related roles *C*) Combined UpSet and volcano plots, with color-coded annotations as in Fig.4*A*, but including additional statistical comparison sets, namely Hypertrophic versus Healthy Donors (HD) and disease-free as well as HD versus disease-free. The absolute frequency of each set is depicted in the adjacent horizontal histogram. The color-coding (‘queries’) of RPPA antibody labels in the volcano plot matches the corresponding colors and sets in the main frequency histogram. *D*) Boxplot with overlaid scattered points of RPPA levels for selected endpoints analyzed in the pivotal and training cohorts and grouped by the sample categories available in each cohort.

**Fig.S8**

*A*) Scatter-plot of RPPA level for ERG analyzed in the pivotal and training cohorts and grouped by available sample category. High (H), Intermediate (I) and Low (L) risk categories were indicated. Blue dash circle represents 65^th^ percentile. The blue dashed line represents hypertrofic/healthy donors basal level in ERG graph. Recurrent and non-recurrent patients within three years are reported. *B*) Scatter-plot of RPPA levels for IL-6 analyzed in pTNM-grouped tumours of the training cohort. *C*) Bivariate scatterplot of RPPA versus PSA levels in individual EV samples analyzed in the training cohort. Color-coding reflects sample category, i.e. HD (green), Hypertrophic (purple) and PCa (red). Per-group density distributions are plotted for the PSA levels (top) as well as for each individual RPPA endpoint (right). *D*) RPPA levels of the top-scoring diagnostic endpoints from the training cohort, grouped by sample type. The color scale maps PSA levels for individual EV samples and dashed lines represent optimal cut-off values obtained from the corresponding ROC curves. *E*) Scatterplots of RPPA levels for selected and significant endpoints in PCa samples from the training cohort, grouped by pTNM. Binary comparisons of TNM groups are reported on each plot (connecting lines) along with statistical significance by Wilcoxon rank sum test. *F*) Bivariate scatterplots of total Met and RANKL RPPA levels versus PSA, with TNM grouping of PCa samples from the training cohort. The pTNM is color-coded as per the legend and the insets display enlarged portions of each plot within the clinically relevant PSA range (5-10 ng/ml).

**Fig.S9**

*A*) Two-way unsupervised hierarchical clustering of RPPA endpoints measured in EVs from a retrospective cohort of 8 low-/intermediate-risk tumors(green), 11 high-risk (orange)and 6 advanced tumours(red). Normalized RPPA intensity values are standardized (Z score) and the heat map color intensity scale indicates high (red), average (black) and low (blue) expression. Cyan line indicates shared antigens among Advanced and high risk cases. *B-C*) Significant antigens expression in the comparison low-/intermediate- vs high-risk or high-risk and advanced tumors analyzed by RPPA assay and with the statistical relevance evaluated by Wilcoxon rank sum test. Representative box plots of selected antigens were reported in (*B*). *D-E*) Graphical representation of mutation frequencies in primary tumours and metastases for selected, highly mutated (*D*, boxplots) and significantly recurrent (*E*, barplot) genes as per in silico elaboration of the publicly available data by Taylor et al. (<http://www.cbioportal.org>; (1)).

**Fig.S10**

*A*) Scatter plots showing RPPA level distribution of c-Myc T58/S62, SHC Y317 and Wnt5a/b antigens analyzed in 8 low-/intermediate-risk tumors, 11 high-risk and 6 advanced tumors. The antigens resulted significant in the statistical comparison reported in Fig.5A.The asterisks indicate the recurrent patients in each set. *B*) Boxplots of normalized RPPA intensity values for selected endpoints (TGF-β, Wnt5a/b, SHC Y317, c-RAF S338, and c-Myc T58/S62 in non-recurrent versus grouped recurrent and advanced tumors (Adv. PCa). Statistical significance of either t test or Wilcoxon rank sum test is reported on each plot and coded with asterisk(s) based on the level of significance (* p<=0.05). *C*) Individual ROC curve analyses of RPPA candidates emerging as significant prognostic markers from statistical comparison of non-recurrent versus recurrent tumours. ROC curve analysis of the minimal number of combined RPPA antigens with values above optimal cut-off allowing sensitive and specific discrimination of patients undergoing advance forms (Combo score). All plots report the AUC value along with the 95% confidence interval as well as p, optimal cut-off, sensitivity and specificity values. *D*) Expression levels of tissue mRNA from the GEO dataset GSE74685 (2)for genes corresponding to prognostic RPPA markers in *B*). Gene expression in bone metastases is compared to (grouped) other type of metastases and statistical significance is reported accordingly (ns not significant, * p<=0.05, ** p<=0.01, *** p<=0.001). *E*) Comparison of expression levels for WNT5A mRNA in primary and metastatic tissues from two different datasets [data source: (1) and GSE32269 (3). WNT5A reached statistical significance (* p <0.05) by unpaired t test in both analyzed datasets. *F)* Expression of ERG mRNA in primary and metastatic tissues stratified for the presence (positive) or absence (negative) of TMPRSS2:ERG-genomic fusion. Data were analyzed from the GEO dataset GSE32269 (3). *G)* ERG mRNA levels in bone *versus* other types of metastasis (grouped) as analyzed in the GEO GSE74685 dataset (2).

**Fig.S11**

*A*) Pathway enrichment analysis of Affymetrix gene expression profiling in 40 PCa primary cell lines (*ex-vivo* collection). The prognostic RPPA actors of pathways scoring as significantly enriched (p value <= 0.01) in the “Bad vs Good” prognosis profiling signature. All selected RPPA candidate genes were upregulated in cells derived from patients with bad prognosis. Indicated GO Terms and scores were generated by Gene Ontology Analysis and Gene Differential expression database (Human U133A Gene ChIP platform, (4, 5)). *B*) Key, selected prognostic RPPA candidates (i.e. TGF-β, Wnt5a/b, Shc1, c-RAF and c-Myc). Modulation of gene expression (i.e. mRNA level in Bad prognosis > Good prognosis group) and corresponding Go Terms in Affymetrix gene profiling when compared to RPPA differentially expressed markers. *C*) Densitometric analyses of Western blots for c-RAF S338, Shc Y317, and c-Myc T58/S62 in lysates of prostate cancer cell lines from *ex vivo* collection described in (*A*). Four samples for each prognosis group (Bad and Good) were used and Hsp90 served as endogenous control. Data were reported as dot plots and annotated with statistical significance (* corresponds to p value <= 0.05) by Student’s t test.

**Supplementary Table legends:**

**Supplementary Table 1 (Table S1)**.

Clinical data of the cohort participants

**Supplementary Table 2 (Table S2).**

Tables represent the list of significantly different (p value < 0.05) RPPA endpoints evaluated in Hypertrofic versus tumor EV samples. Statistical comparisons were performed by means of Wilcoxon rank sum test and p values reported were corrected for multiple comparisons by FDR method. Grey scale colors represent common antigens.

**Supplementary Table 3(Table S3).** Tables represent the list of significantly different (p value < 0.05) RPPA endpoints evaluated in or Disease free (DF) versus tumour EV samples. Statistical comparisons were performed by means of Wilcoxon rank sum test and p values reported were corrected for multiple comparisons by FDR method. Grey scale colors represent common antigens.

**Supplementary Table 4(Table S4).** Tables represent the list of significantly different (p value < 0.05) RPPA endpoints evaluated in healthy donors (HD) versus tumour EV samples. Statistical comparisons were performed by means of Wilcoxon rank sum test and p values reported were corrected for multiple comparisons by FDR method. Green and red color-coding indicates up- and down-regulation, respectively, in tumour samples.

**Supplementary Table 5(Table S5).**

Clinical data of fifteen year follow up patients grouped in the risk assessment cohort.

**References**

1. Taylor BS, Schultz N, Hieronymus H, Gopalan A, Xiao Y, Carver BS, et al. Integrative genomic profiling of human prostate cancer. Cancer cell. 2010;18(1):11-22.

2. Haider M, Zhang X, Coleman I, Ericson N, True LD, Lam HM, et al. Epithelial mesenchymal-like transition occurs in a subset of cells in castration resistant prostate cancer bone metastases. Clinical & experimental metastasis. 2016;33(3):239-48.

3. Cai C, Wang H, He HH, Chen S, He L, Ma F, et al. ERG induces androgen receptor-mediated regulation of SOX9 in prostate cancer. The Journal of clinical investigation. 2013;123(3):1109-22.

4. Nanni S, Aiello A, Re A, Guffanti A, Benvenuti V, Colussi C, et al. Estrogen-dependent dynamic profile of eNOS-DNA associations in prostate cancer. PloS one. 2013;8(5):e62522.

5. Nanni S, Priolo C, Grasselli A, D'Eletto M, Merola R, Moretti F, et al. Epithelial-restricted gene profile of primary cultures from human prostate tumors: a molecular approach to predict clinical behavior of prostate cancer. Molecular cancer research : MCR. 2006;4(2):79-92.
