# Supplementary figures and images for: Diagnostic and prognostic potential of the proteomic profiling of serum-derived extracellular vesicles in prostate cancer
Source: Cell Death Dis. 2021 Jun 21;12(7):636. doi: 10.1038/s41419-021-03909-z (PMC8215487; doi:10.1038/s41419-021-03909-z)

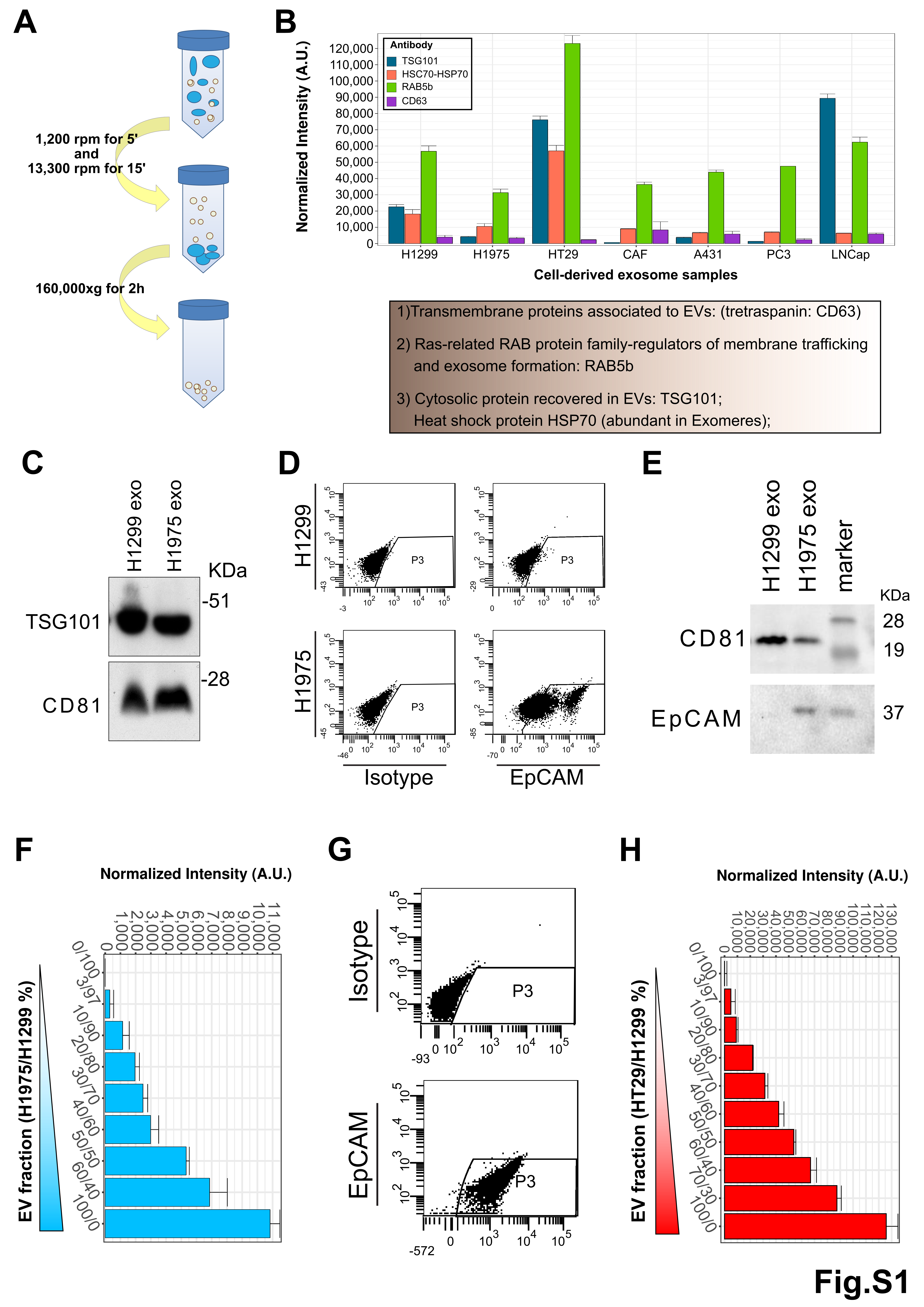

Supplement: Supplementary file 3 — Supplementary Fig.S1 [file 41419_2021_3909_MOESM3_ESM.png]

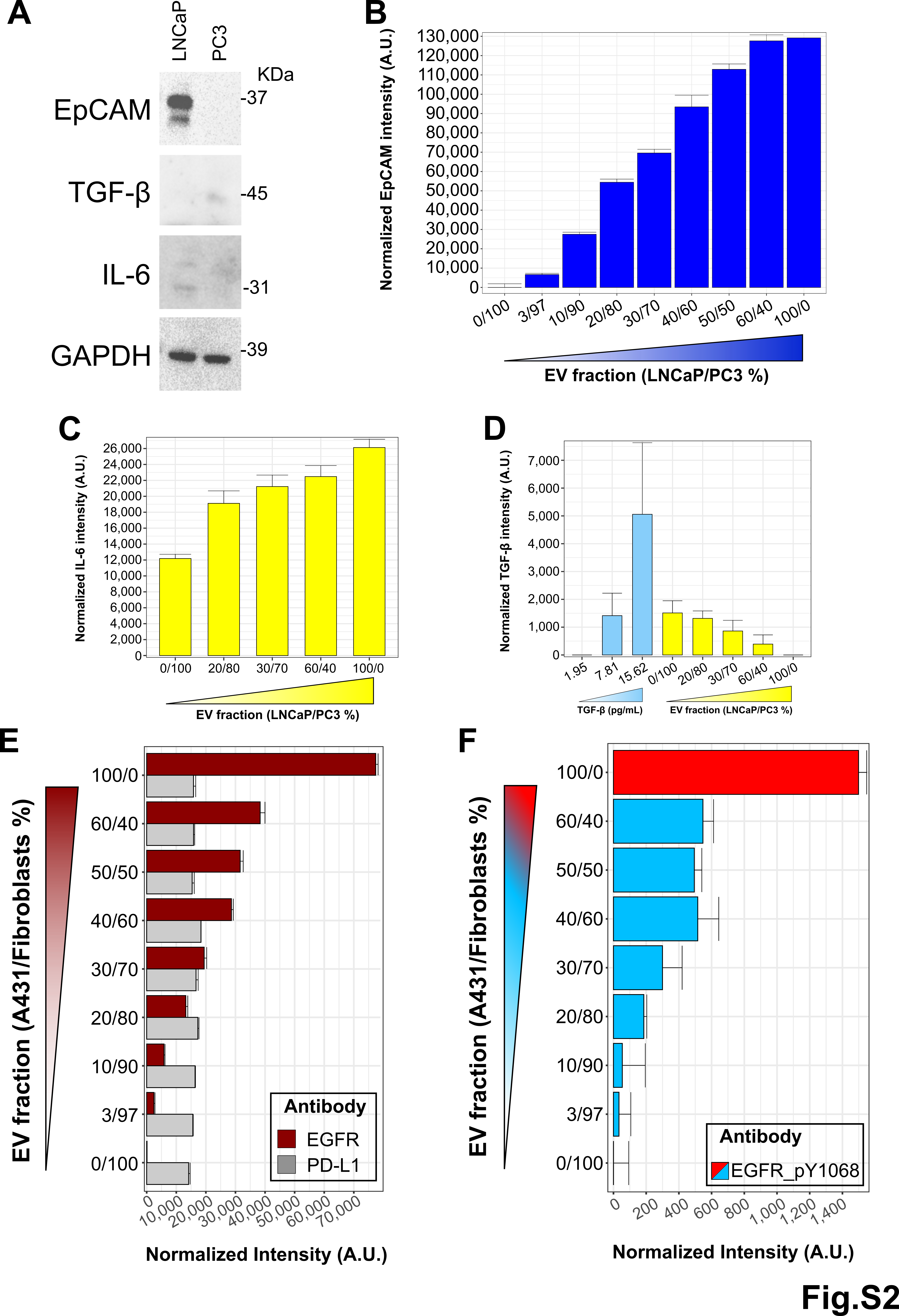

Supplement: Supplementary file 4 — Supplementary Fig.S2 [file 41419_2021_3909_MOESM4_ESM.png]

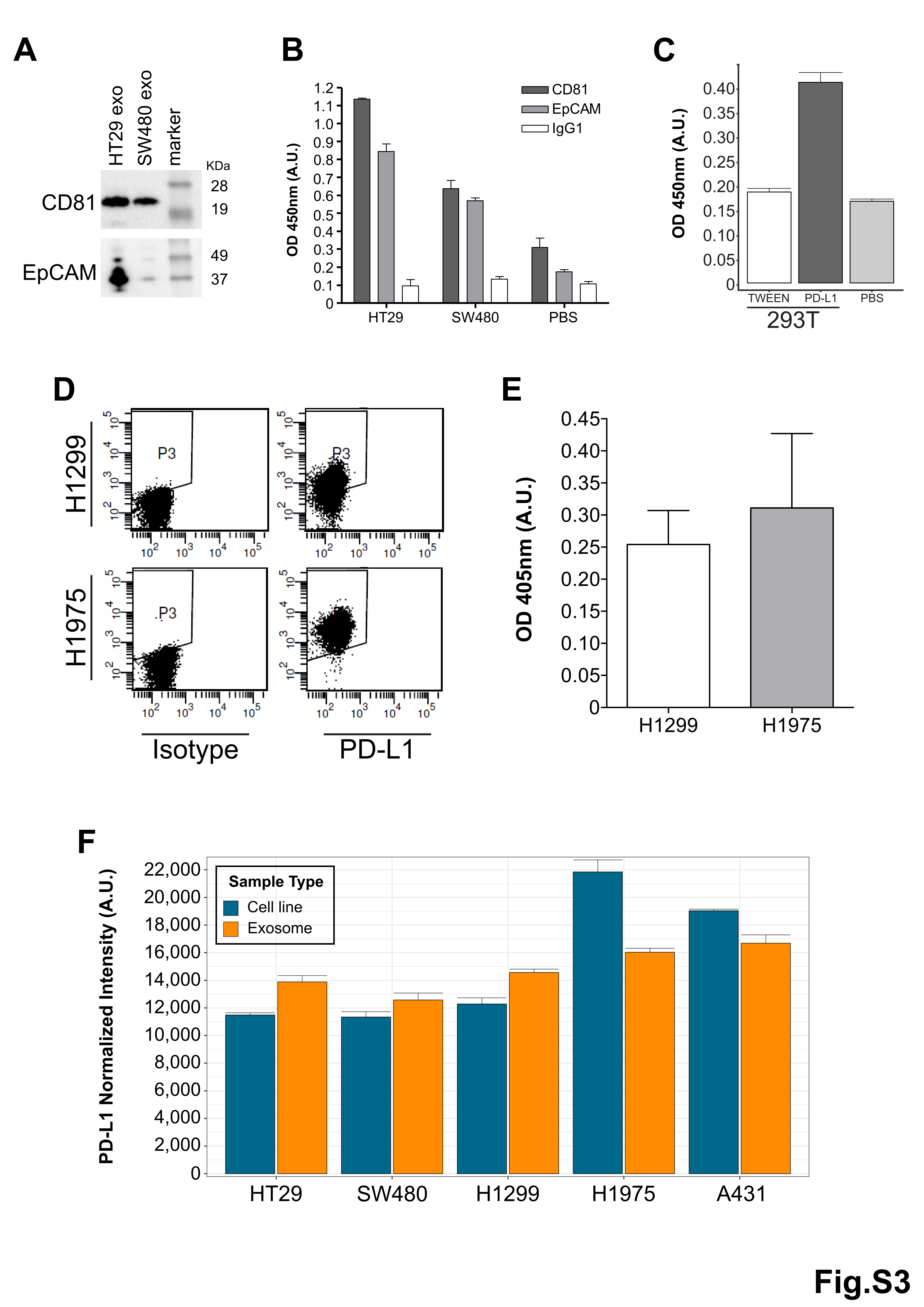

Supplement: Supplementary file 5 — Supplementary Fig.S3 [file 41419_2021_3909_MOESM5_ESM.png]

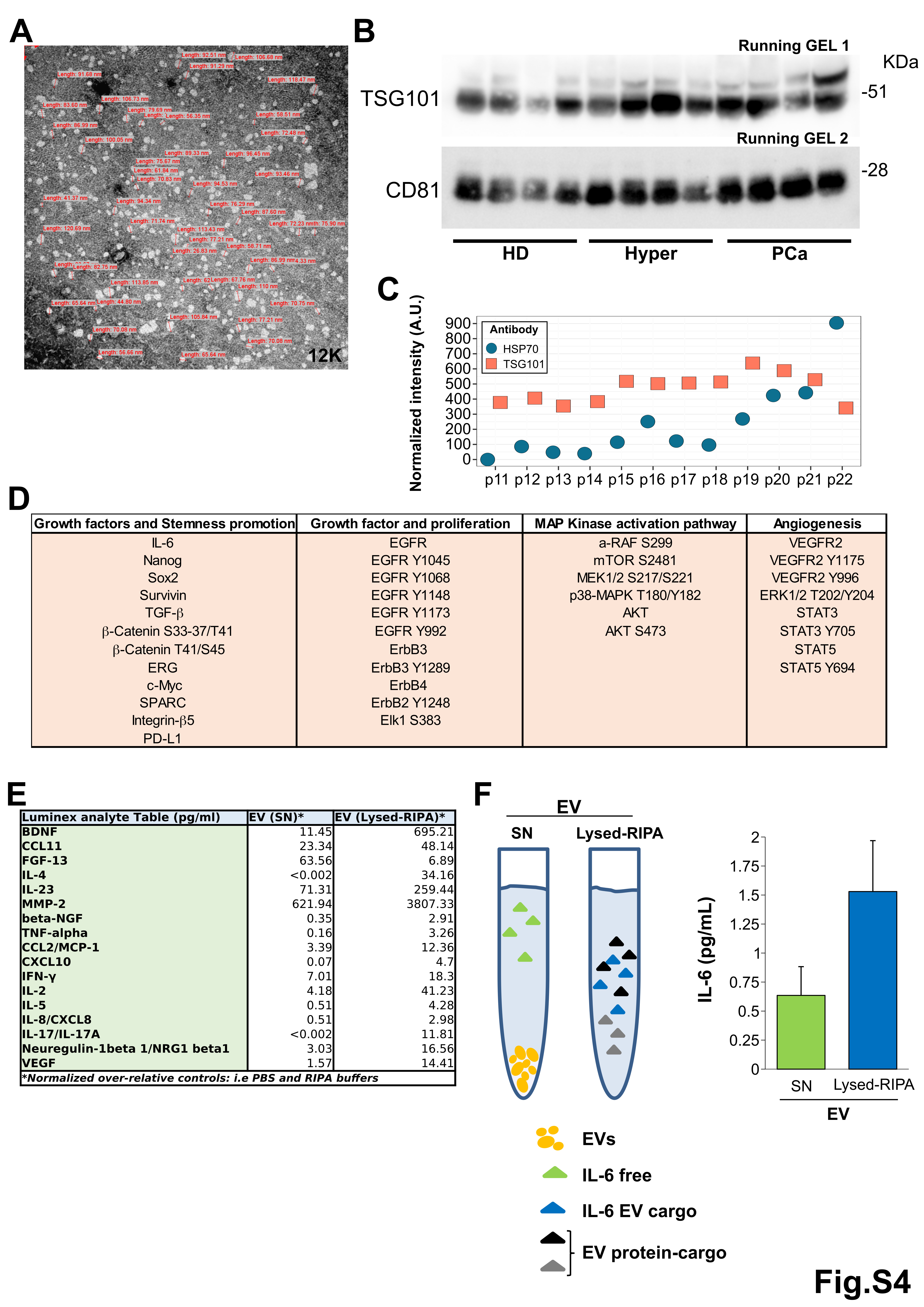

Supplement: Supplementary file 6 — Supplementary Fig.S4 [file 41419_2021_3909_MOESM6_ESM.png]

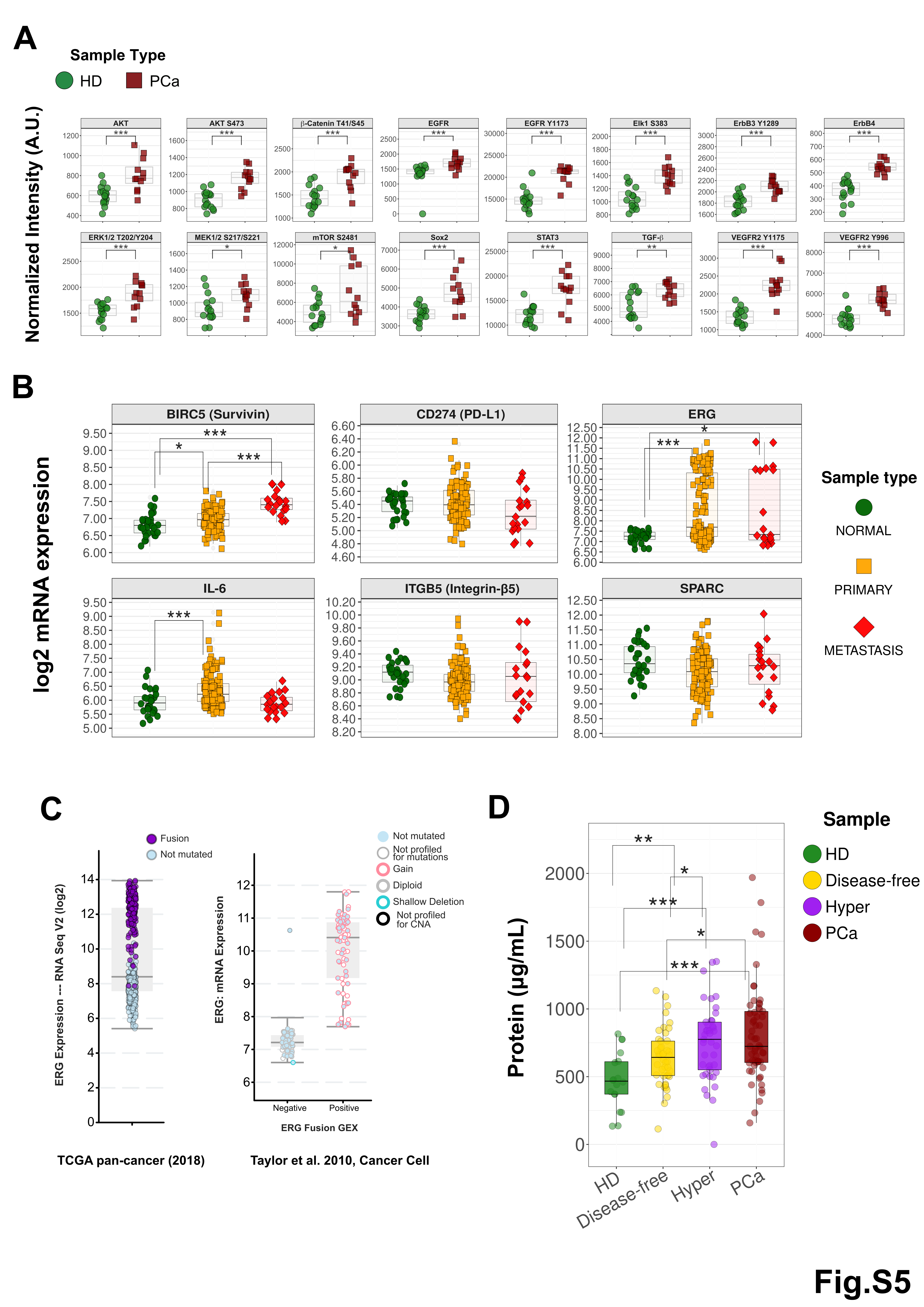

Supplement: Supplementary file 7 — Supplementary Fig.S5 [file 41419_2021_3909_MOESM7_ESM.png]

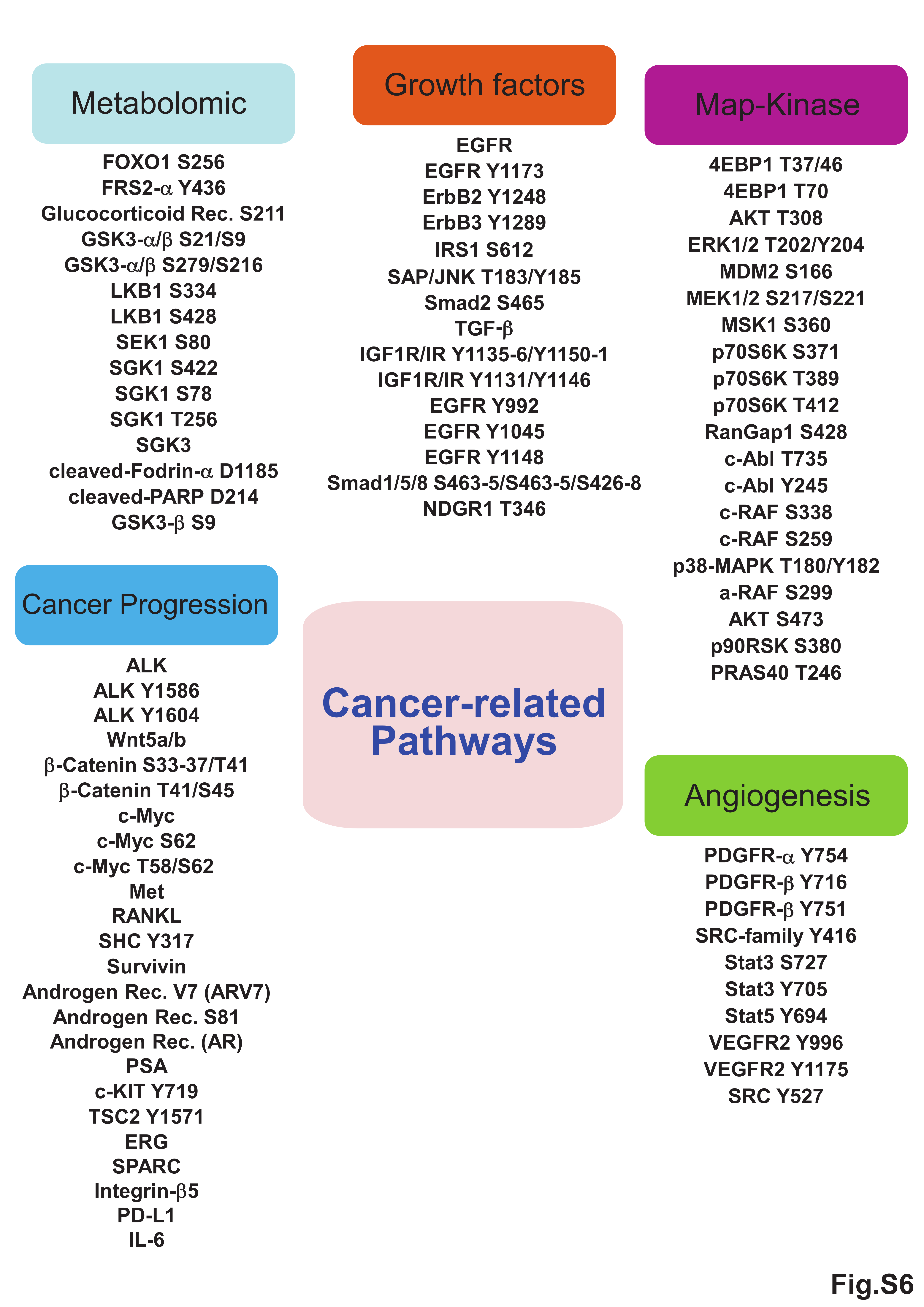

Supplement: Supplementary file 8 — Supplementary Fig.S6 [file 41419_2021_3909_MOESM8_ESM.png]

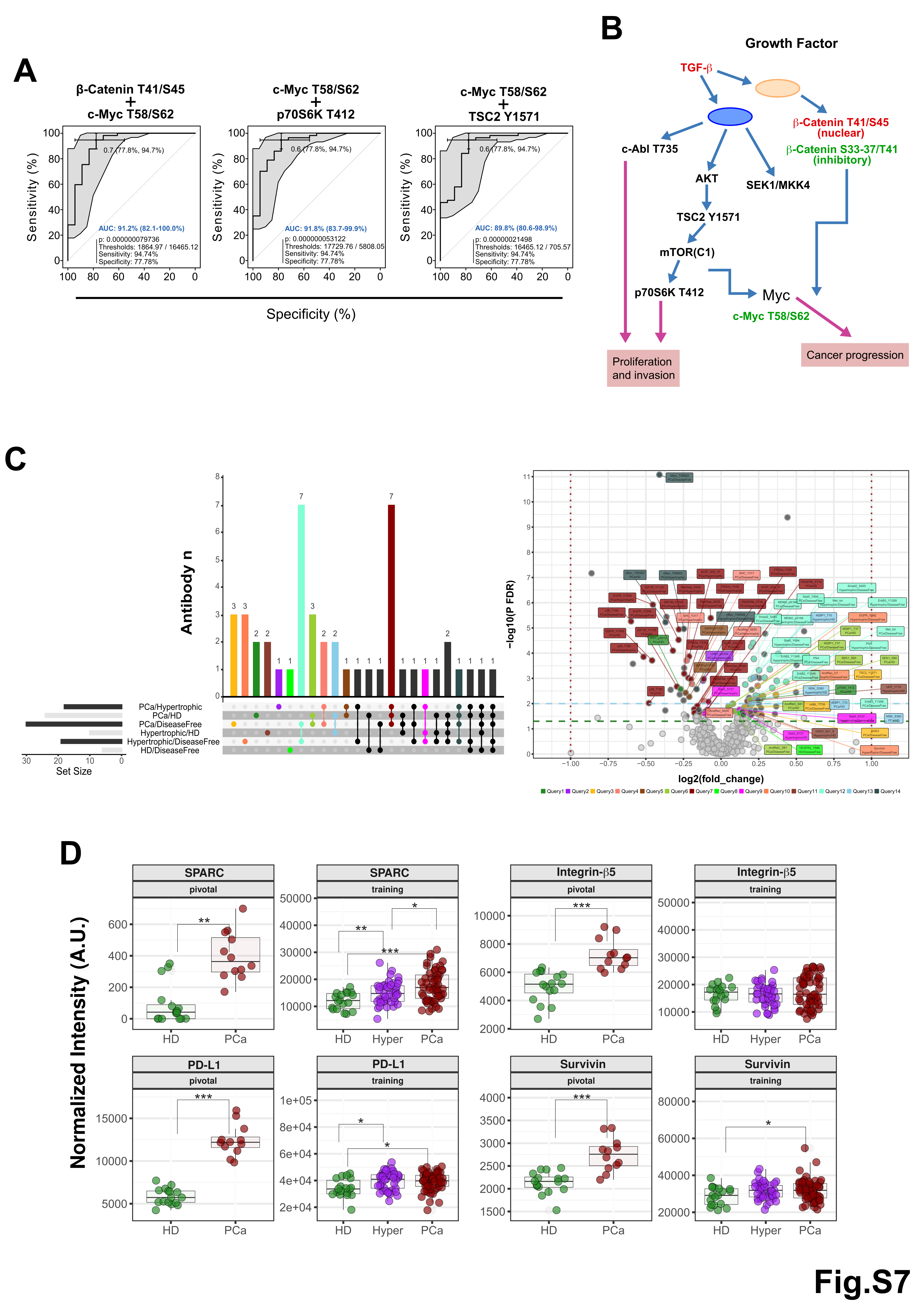

Supplement: Supplementary file 9 — Supplementary Fig.S7 [file 41419_2021_3909_MOESM9_ESM.png]

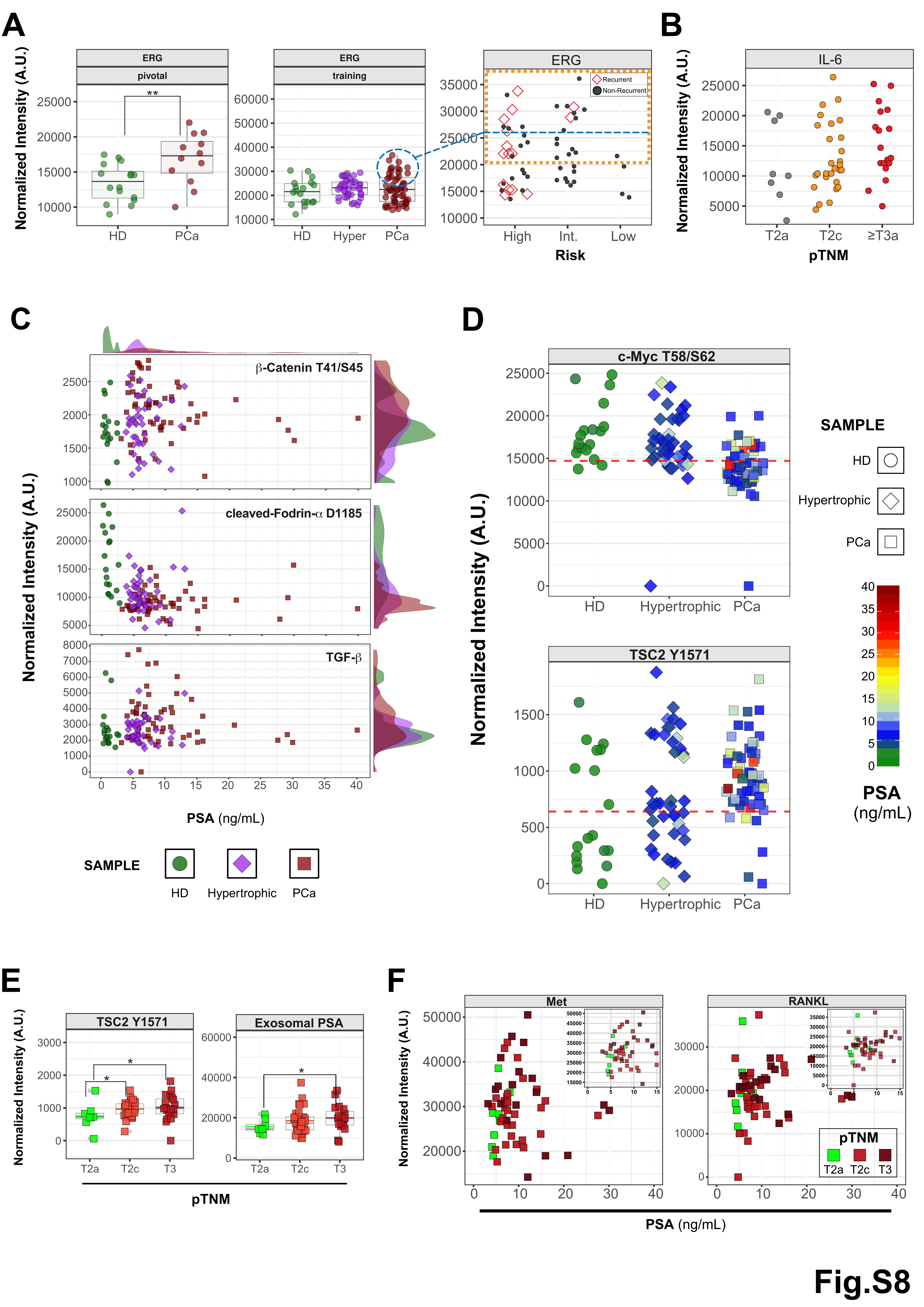

Supplement: Supplementary file 10 — Supplementary Fig.S8 [file 41419_2021_3909_MOESM10_ESM.png]

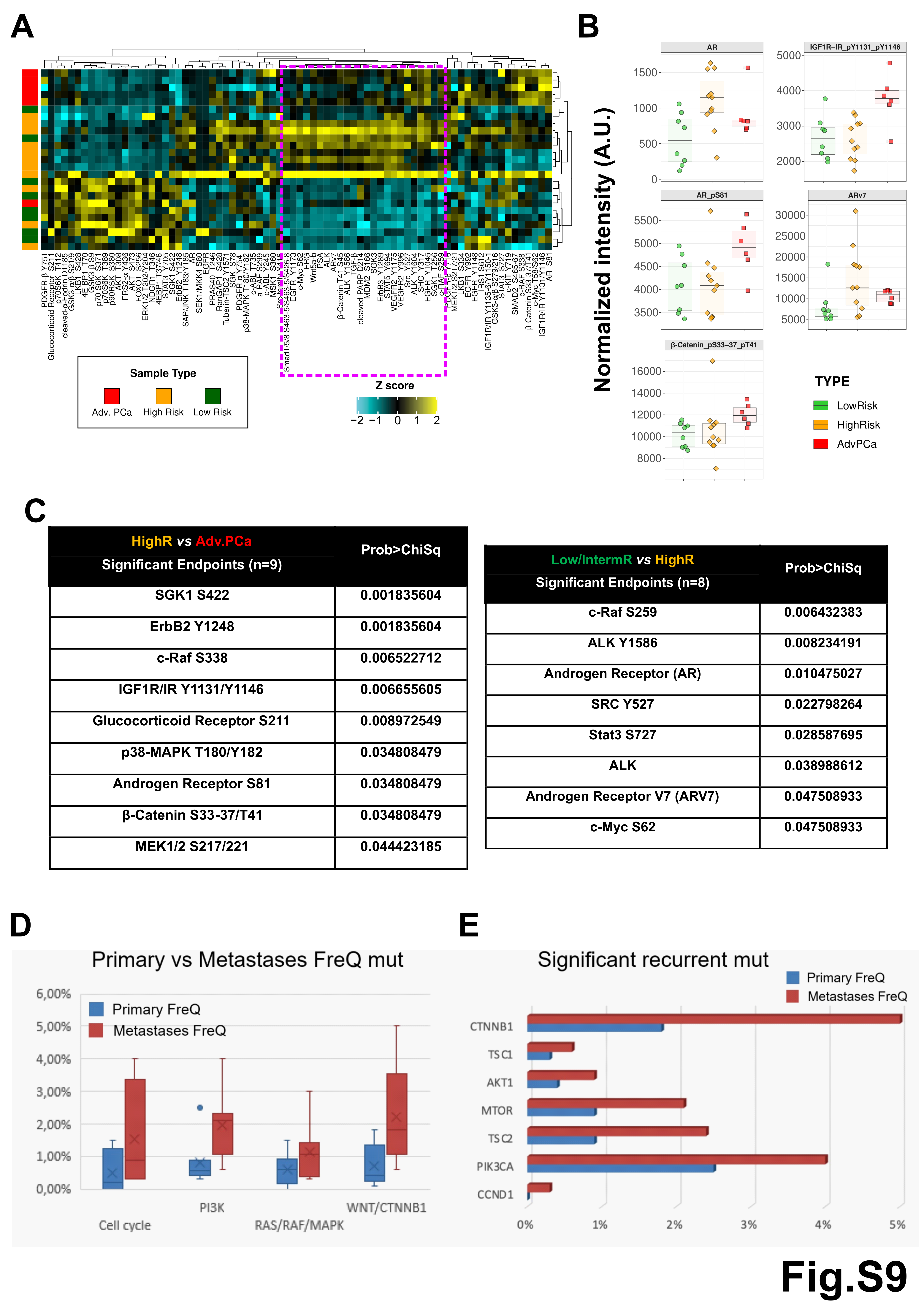

Supplement: Supplementary file 11 — Supplementary Fig.S9 [file 41419_2021_3909_MOESM11_ESM.png]

**A**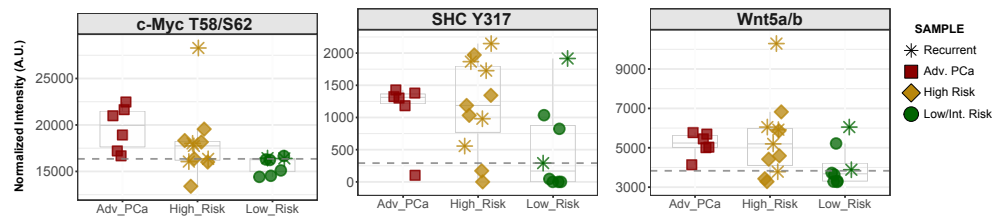**B**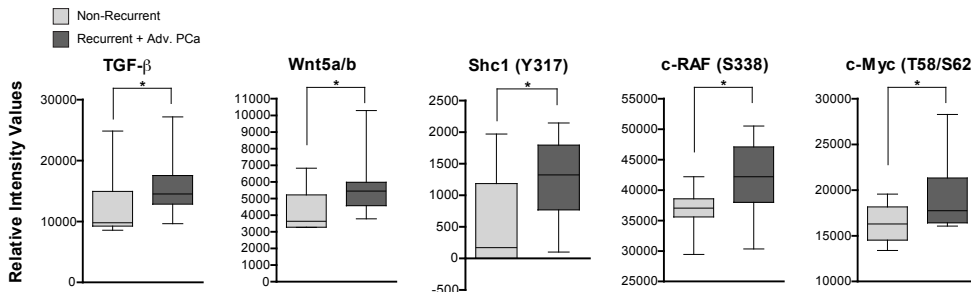**D**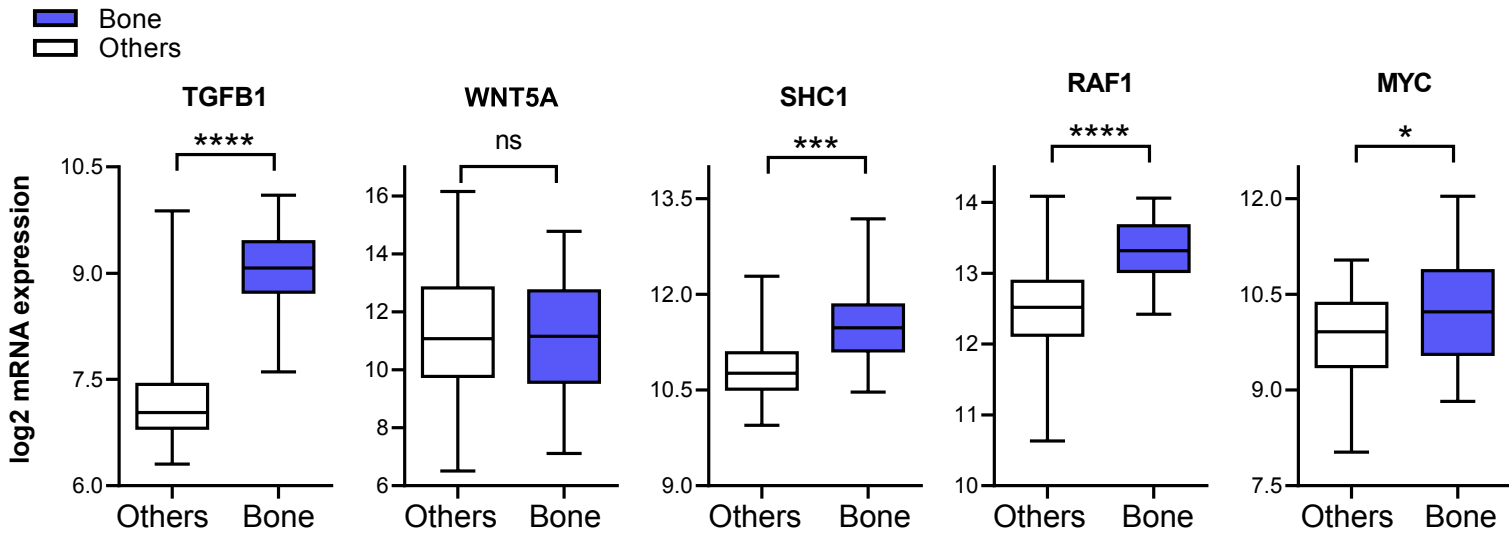**E**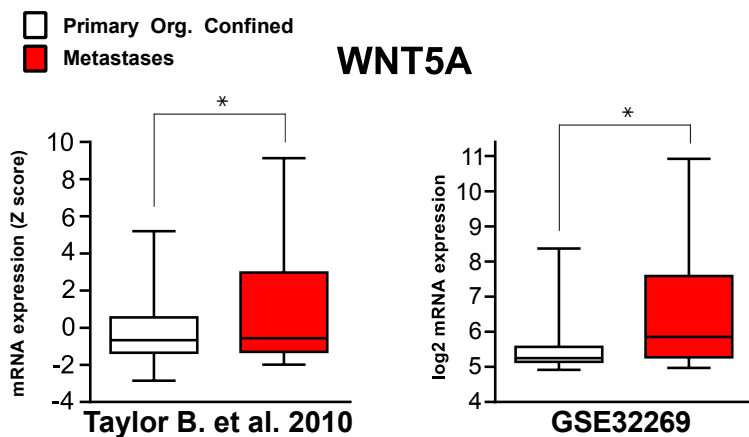**F**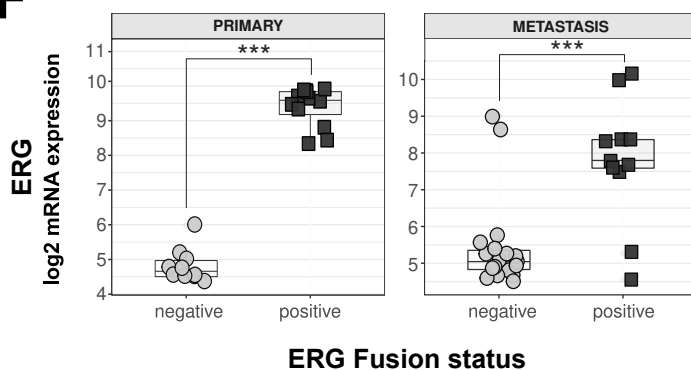**G**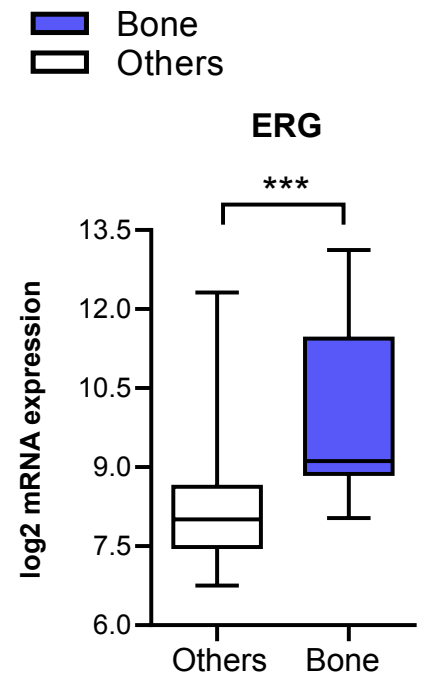**Fig.S10**

Supplement: Supplementary file 12 — Supplementary Fig.S10 [file 41419_2021_3909_MOESM12_ESM.pdf]

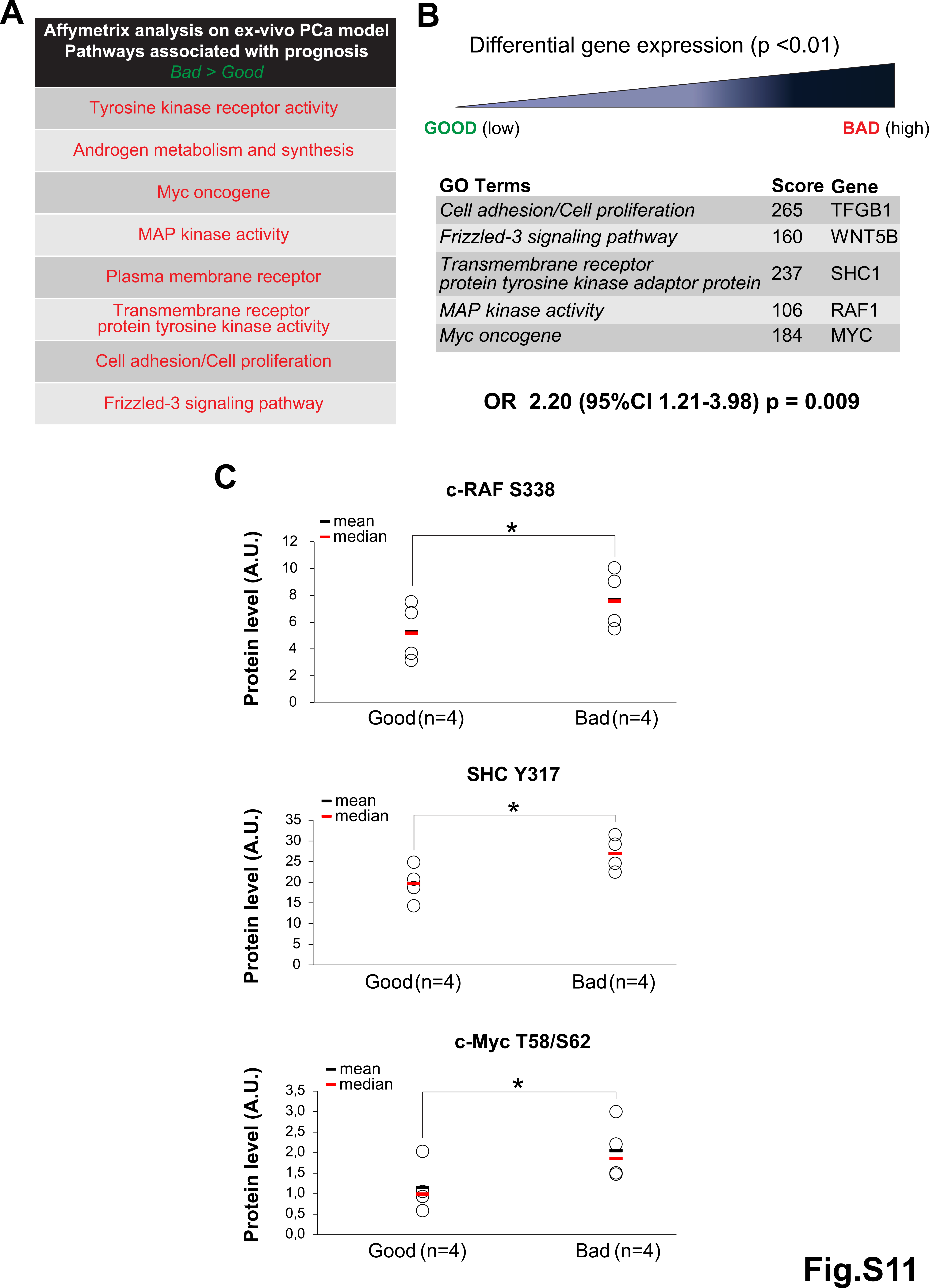

Supplement: Supplementary file 13 — Supplementary Fig.S11 [file 41419_2021_3909_MOESM13_ESM.png]
